# Preparation of a Key Intermediate En Route to the Anti-HIV Drug Lenacapavir

**DOI:** 10.1021/acs.joc.3c02855

**Published:** 2024-03-06

**Authors:** Juan C. Caravez, Yuting Hu, Erfan Oftadeh, Kirubel T. Mamo, Bruce H. Lipshutz

**Affiliations:** Department of Chemistry & Biochemistry, University of California, Santa Barbara, CA 93106, United States

## Abstract

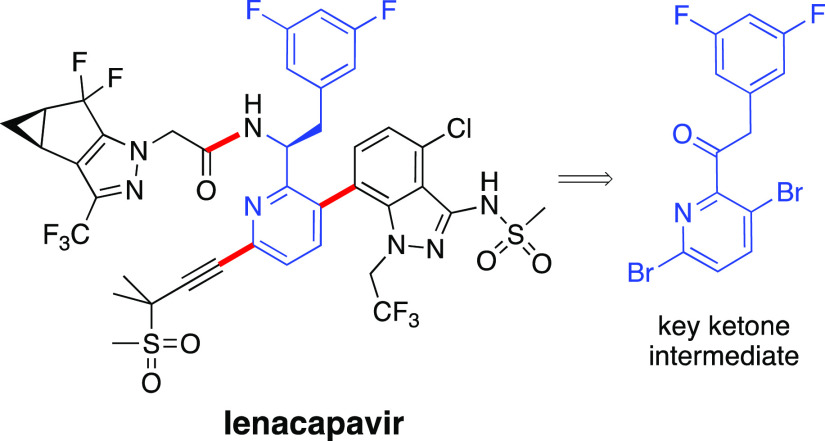

A very efficient four-step synthesis of the main fragment of Gilead’s anti-HIV drug lenacapavir is described. The route showcases a 1,2-addition to an intermediate aldehyde using an organozinc halide derived from a commercially available difluorobenzyl Grignard reagent. This sets the stage for the oxidation of the resulting secondary alcohol to the desired ketone, which relies solely on catalytic amounts of TEMPO together with NaClO as the terminal oxidant, affording the targeted ketone in 67% overall yield.

## Introduction

**Scheme 1:**
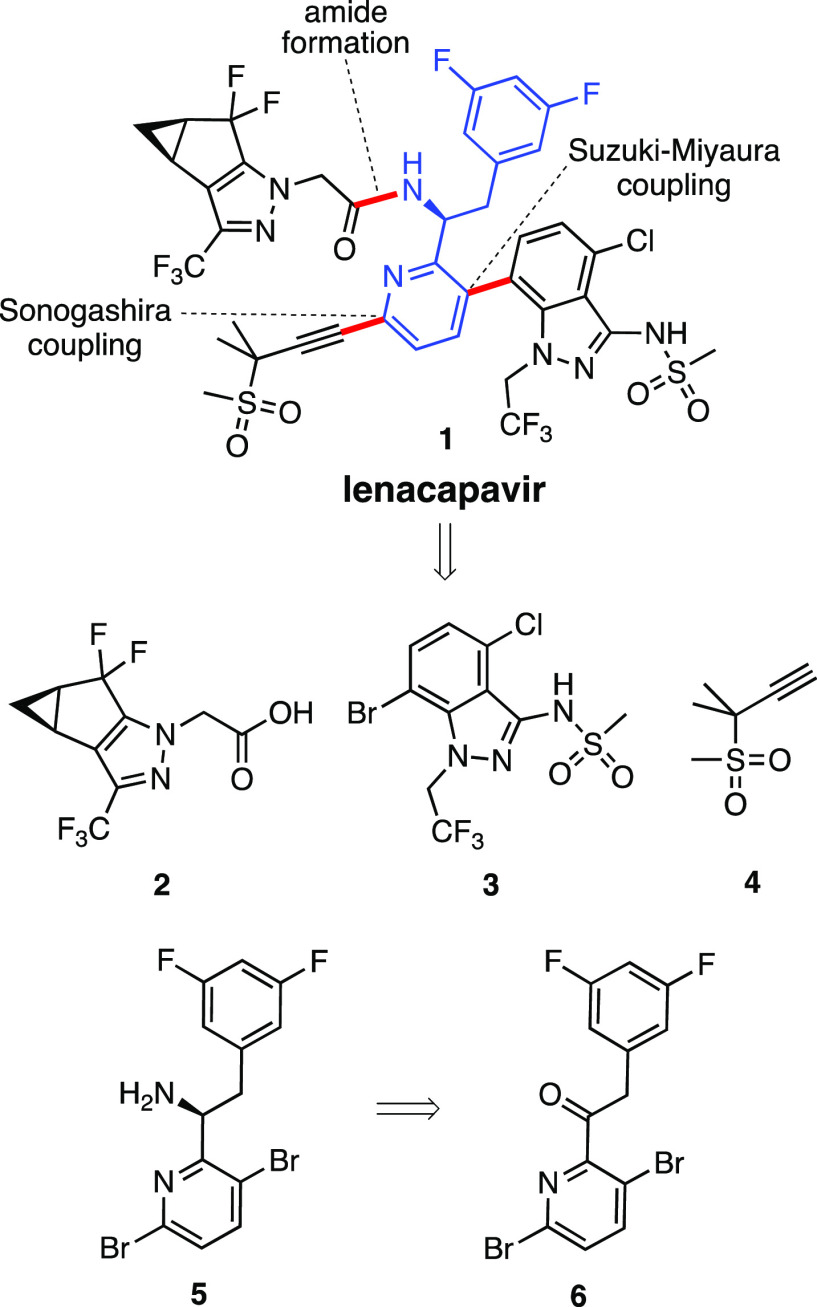
Key Bonds Associated with Any Route to Lenacapavir

The human immunodeficiency virus (HIV) and its progression to acquired immunodeficiency syndrome (AIDS)^1^ have claimed the lives of millions of people worldwide since the early 1980s.^2^ In 2022, the FDA approved Gilead’s lenacapavir (Scheme 1), sold as Sunlenca. Lenacapavir is a potent two-dose treatment given only once a year for HIV-1-positive patients, found to be up to 83% effective in patient improvement.^3^ This drug functions as a capsid inhibitor preventing the virus from reproducing, a novel mechanism of action for this type of treatment.^4^ Given the effectiveness and, hence, anticipated impact of lenacapavir, accessibility by those in low- and middle-income countries has become a high priority. This translates into cost, an issue that can be accommodated in large measure by the synthetic route used to produce this drug. Retrosynthetic analysis indicates that lenacapavir is composed of four main fragments (Scheme 1).^5^ Intermediate **6** represents a highly functionalized precursor to the core of the molecule to which the additional components can be introduced via (1) a Sonogashira reaction associated with the alkyne **4**, (2) a Suzuki–Miyaura coupling that inserts the benzpyrazole moiety **3**, and (3) an amide-forming process for the insertion of the nonracemic carboxylic acid **2**.

**Scheme 2:**
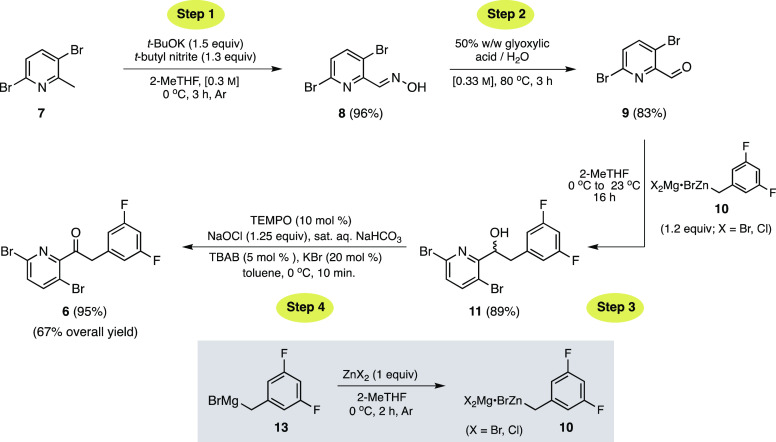
Four-Step Synthesis of Ketone **6**

Efforts were set toward developing a cost-effective synthesis of ketone **6**, a likely precursor to **5**, as an alternative route ultimately to lenacapavir. To the best of our knowledge, there is no openly available route to ketone **6**. Herein, we describe a novel four-step sequence to this key, central component of lenacapavir that proceeds in 67% overall yield (Scheme 2).

## Results and Discussion

**Scheme 3:**
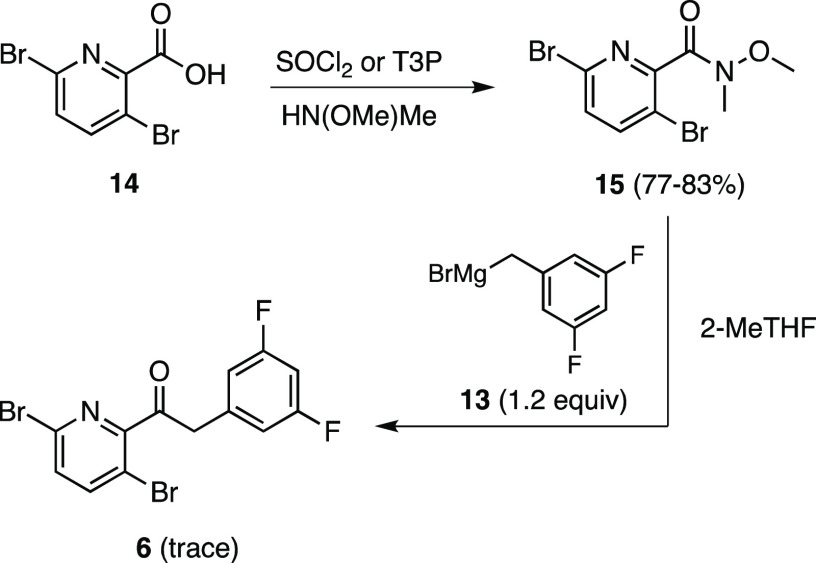
Synthesis of Ketone **6** via Weinreb Amide **15**

Initial efforts focused on the use of 3,6-dibromopicolinic acid (**14**) as the starting material, en route to Weinreb amide **15**, anticipating the addition of commercially available 3,5-difluorobenzylmagnesium bromide (**13**) to afford ketone **6** in a two-step fashion (Scheme 3).^6,7^ Weinreb amide **15** was prepared in moderate yields of 77 and 83% using thionyl chloride^8^ and T3P,^9^ respectively. Introduction of commercially available Grignard **13** in 2-MeTHF, however, led to only trace amounts of ketone **6**. Aside from the lack of efficiency observed in this transformation, and since the cost of acid **14** is quite high, this route was abandoned in favor of a far more attractive sequence using the corresponding picoline analogue **7** (Table 1).^10^

**Table 1:**
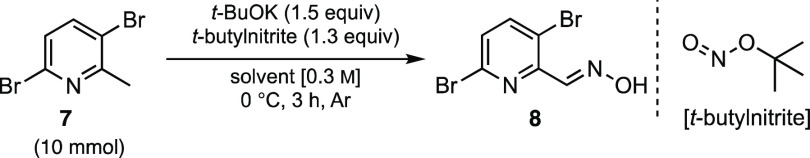
Optimization of Step 1

entry	KO*t*-Bu (equiv)	addition order	temperature	yield^a^
1^d^	1.0	KO*t*Bu, then *t*-bultylnitrite	0 °C to rt	NR
2^d^	1.5	KO*t*Bu, then *t*-bultylnitrite	0 °C to rt	11%
3^d^	1.5	*t*-bultylnitrite, then KO*t*Bu dropwise	0 °C to rt	45%
4^d^	1.5	*t*-bultylnitrite, then KO*t*Bu dropwise	keep at 0 °C	93%
**5**^e^	**1.5**	*t***-bultylnitrite, then KO***t***Bu dropwise**	keep at 0 °C	**94%**^b^
**6**^e^	**1.5**	*t***-bultylnitrite, then KO***t***Bu dropwise**	keep at 0 °C	**96%**^c^

a Isolated yield.

b Run on a 40 mmol scale.

c Run on a 20 mmol scale.

d THF was used as solvent.

e 2-MeTHF was used as solvent.

Starting from inexpensive commercially available 3,6-dibromo-2-methylpyridine **7**, oximation cleanly leads to oxime **8** in excellent yield (96%) using recyclable 2-MeTHF.^11^ Hydrolysis of **8** in the presence of 50 wt % glyoxylic acid effectively generates aldehyde **9** (Scheme 2). 1,2-Addition of the derived zinc halide **10** generated from benzylic Grignard **13** in 2-MeTHF^11^ to aldehyde **9** led to alcohol **11**. Finally, the oxidation of **11** to ketone **6** was smoothly accomplished in minutes at 0–5 °C using catalytic TEMPO and a slight excess of NaClO in a biphasic mixture (Scheme 2).

A two-step synthetic route was employed to furnish aldehyde **9**, following a literature procedure.^5^ Initial screening of the oximation of **7** in anhydrous THF in the presence of *t*-butylnitrite (TBN) and potassium *t*-butoxide as the base (1 equiv) at 0 °C to rt resulted in no reaction (Table 1, entry 1). Increasing the amount of base to 1.5 equiv led to some of the desired product, albeit in poor yield (ca. 11%; entry 2). However, by changing the order of addition a far better reaction resulted (entry 3). That is, adding **7** along with TBN in THF followed by the dropwise addition of a solution of *t-*BuOK in THF increased the yield to about 45% (entry 3). Finally, it was found that not only a dropwise addition of the base but also maintaining the temperature at 0 °C was crucial (entries 4–6). Both THF and 2-MeTHF performed equally well; however, we opted to carry out this transformation in 2-MeTHF, which is a preferred green solvent.^11^ Upon quenching this reaction with sat. aqueous NH_4_Cl, the solvent can easily be recovered to the extent of 85–89% and then reused (SI, S5). The crystalline nature of oxime **8** allowed for simple filtration, followed by several water washes, after which the material could be used in the next step (SI, S5). Several acids were then screened under aqueous conditions (SI, Table S2). Ultimately, oxime **8** was best hydrolyzed using 50% w/w glyoxylic acid/H_2_O at 80 °C over 3 h, leading to aldehyde **9** in 83% yield. This hydrolysis leading to **9** required only a simple filtration, followed by water washes to obtain relatively pure material (99% pure, by ^1^H NMR).

Alcohol **11** was anticipated to form via a 1,2-addition of Grignard **13**. Optimization revealed two major side products **18** and **19**, the former being favored (Table 2, entries 1 and 2). Due to the high reactivity of Grignard **13** in combination with the highly activated position-6 on aldehyde **9**, a competing S_N_Ar reaction led to byproduct **18**. To minimize this undesired material, temperature, stoichiometry, and order of addition were probed (see the SI, Table S3) with little improvement observed. To increase the electrophilicity of the carbonyl carbon, a Lewis acid (BF_3_•OEt_2_) was introduced; however, only 56% conversion was noted of which 50% selectivity for the 1,2-addition product was formed along with 38% of the S_N_Ar product and 11% of alcohol **19** (entry 5).

**Table 2:**
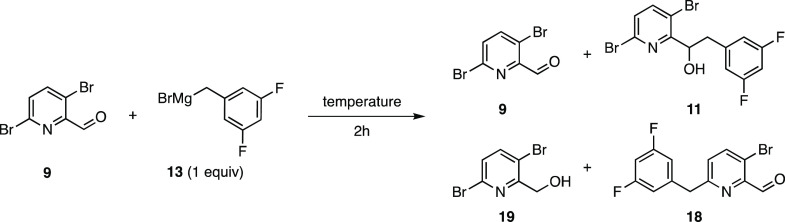
Optimization Studies Leading to Product **11**

entry^a^	temperature	conditions	9 (%)^c^	11 (%)^c^	18 (%)^c^	19 (%)^c^
1	0 to 23 °C		11	34	44	3
2	keep at 0 °C		9	38	40	6
3	keep at 0 °C	aldehyde:Grignard = 1:12	5	14	66	3
4	keep at 0 °C	add Grignard dropwise over 2 h	42	10	33	5
5	keep at 0 °C	add 1 equiv BF_3_OEt_2_	44	27	20	6
6	keep at 0 °C	mix 1 equiv CuCN with Grignard, then use 0.8 equiv	12	43	36	6
7	keep at 0 °C	use organozinc reagent made from bromide and zinc dust	11	60	27	nd
8	0 to 23 °C	mix 1 equiv ZnBr_2_ with Grignard, then use 1 equiv mixture, 12 h	15	80	3	2
9	0 to 23 °C	mix 1 equiv ZnBr_2_ with Grignard, then use 1.2 equiv mixture, 12 h	8	88	2	1
10^d^	0 to 23 °C	mix 1 equiv ZnBr_2_ with Grignard, then add solution of **9** dropwise, 16 h		89^b^		

a Reactions run on a 0.2 mmol scale.

b Isolated yield.

c Yield determined by GC-MS using naphthalene as an internal standard.

d Reaction was run on a 0.25 mmol scale. For experimental conditions, see the SI, S8.

Among efforts to decrease side product formation (**18** and **19**), the use of CuCN and zinc salts were added to Grignard **13** to form the less reactive cyanocuprate and zinc halide species, respectively. The use of CuCN was ineffective, leading to only 43% of **11** along with 36% of **18** and 6% of **19** (entry 6). The organozinc reagent was prepared from zinc dust and difluorobenzyl bromide. However, 27% of side product **18** was obtained (entry 7). Interesting, however, is that side product **19** was not observed. A screening of zinc salts (e.g., ZnBr_2_) added to Grignard **13** gratifyingly resulted in a decrease in the formation of **18** (<6%) and only ca. 2–3% of **19**.^12^ This indicated that the zinc halide complex should be generated by adding one equivalent of zinc salt to a solution of benzyl Grignard **13** in 2-MeTHF. This new solution was then used to selectively carry out 1,2-addition onto aldehyde **9** generating alcohol **11**. When performed in this fashion, an 88% yield of **11** was obtained while minimizing side product formation (entry 9). Furthermore, in efforts to lower the overall cost, use of ZnCl_2_ in place of ZnBr_2_ showed similar results (89%; entry 10).^13^

**Scheme 4:**
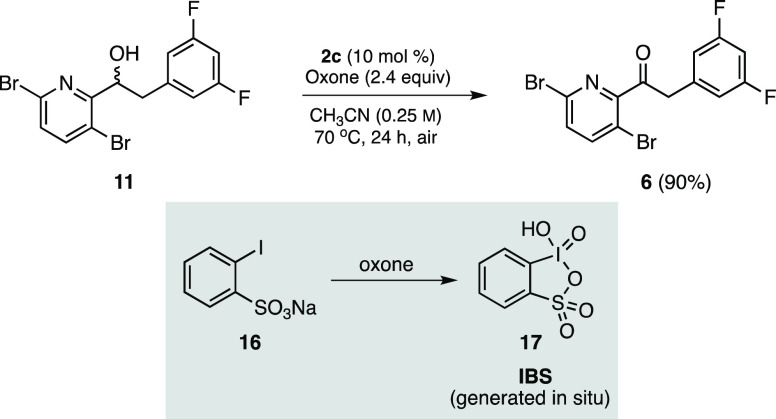
Initial Oxidation of **11** to Ketone **6**

**Figure 1:**
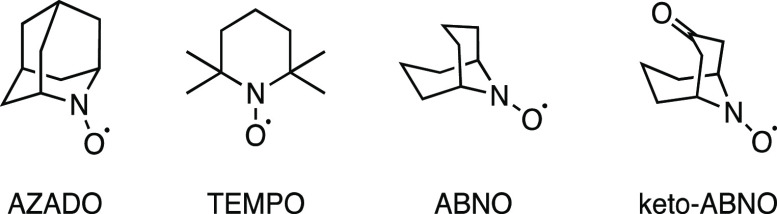
Commonly used nitroxyl radical catalysts.

Once alcohol **11** was in hand, attention turned toward finding an efficient oxidation en route to ketone **6**. The first focused on stoichiometric amounts of a hypervalent iodide species^14^ notwithstanding their potentially explosive nature.^15^ In our search (see the SI, Table S4), the majority of reagents and conditions screened led to little-to-no conversion. Eventually, using commercially available sodium 2-iodobenzenesulfonate **16** (10 mol %) along with Oxone^16^ in acetonitrile (generating IBS **17** in situ) gave **6** in 90% yield^17^ (Scheme 4). Despite the efficiency of this transformation, we envisioned that an even more facile, green, and inexpensive method could be found. Further study led to the evaluation of nitroxyl radical catalysts (Figure 1), as these are well-known for the oxidation of a wide range of primary and secondary alcohols.^18,19^ Among these *N*-oxyl catalysts, 2,2,6,6-tetramethylpiperidinyloxyl (TEMPO) stands out as the most available and likely the most cost-effective option. However, the presence of bulky groups (i.e., tetramethyl) adjacent to the active site suggested that steric effects might lower its catalytic activity, particularly in the case of hindered alcohols.^18^ Consequently, alternative *N*-oxyl catalysts have emerged in the past decade mainly aiming to overcome this potential barrier.^18,20^

Preliminary studies using *N*-oxyl radical catalysts for this oxidation involved screening based on the steric factors present in these systems. Both AZADO and ABNO were examined, initially leading to no reaction in most cases. Using a hypervalent iodine species such as diacetoxyiodobenzene (PIDA) or oxygen gas as the stoichiometric oxidant in combination with either TEMPO or AZADO, the latter in combination with sodium hypochlorite (NaClO) in a biphasic solvent system (H_2_O:CH_2_Cl_2_) gave some oxidation. Worth noting is the crucial role temperature plays in this transformation. That is, for temperatures of 22 °C and above, no product formation was observed even with relatively high loadings of catalyst. Furthermore, in addition to maintaining the temperature at 0 °C, the reaction time required close monitoring. When this transformation was carried out at 0 °C for a period of 1 h, only 40% product was observed via qNMR. But when the reaction time was reduced to ca. 8 min, a 70% yield of ketone **6** was observed (also by qNMR). Apparently, this transformation is much more efficient at colder temperatures (ca. 0 °C), likely due to the short lifetime of in situ-generated hypobromous acid and the instability of the oxonium species containing the *N*-oxyl catalyst at room temperature.^18^ Extended reaction times and amount of oxidant presumably lead to overoxidation and/or decomposition of **6** (see the SI, Table S5).

Continued investigation indicated that reducing the amount of NaClO could minimize side product formation. A thorough screening of oxidant was carried out, confirming the existence of products of overoxidation/decomposition, thereby decreasing the resulting yield of **6** (see the SI, Table S6). Attempts were then made to find an alternative, potentially recoverable solvent and avoid using the common and generally accepted organic solvent for this type of oxidation such as environmentally egregious CH_2_Cl_2_.^21^ Screening solvents led to the potential for toluene and ethyl acetate to serve as good replacements for CH_2_Cl_2_ (see the SI, Table S7). Reoptimization of the loading of NaClO using toluene (see the SI, Table S8) indicated that approximately 1.25 equiv of a 10–15% aq. NaClO in 0.2 M toluene led to a nearly quantitative yield in 10 min at 0 °C, with 97% purity by HPLC without any further purification (Table 3, entry 1).

**Table 3:**
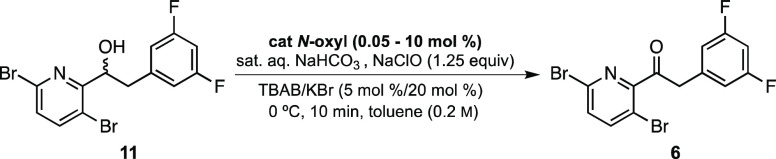
Screening of Various *N*-Oxyl Catalysts

entry^e^	catalyst	mol %	conversion of **11**^a^	**6** (%)^b^	side product(s) (%)^c^
1^d^	AZADO	1	99	97	2
2	AZADO	0.5	99	93	6
3	AZADO	0.25	96	95	1
4	ABNO	1	99	98	1
5	keto-ABNO	5	52	32	20
**6**	**TEMPO**	**10**	**98**	**95**	**3**

a Consumption of **11** based on HPLC.

b Conversion of **11** to **6** based on HPLC.

c Conversion of **11** to side product(s) based on HPLC analysis.

d Run on a 0.25 mmol scale.

e Run on a 0.1 mmol scale.

Finally, optimization of the loading of the organocatalyst remained. Screening this reaction variable showed that only 0.25 mol % of highly active AZADO is required for an efficient oxidation of **11** to **6** (Table 3, entry 3). Other catalysts, such as ABNO, showed similar efficiency to that seen with AZADO (entry 4). However, 9-azabicyclo[3,3,1]nonan-3-one-9-oxyl (keto-ABNO) was not as active as ABNO (entry 5).

Although initial attempts using TEMPO were unsuccessful, this far less costly catalyst was revisited under our newly optimized conditions. The use of 10 mol % TEMPO now gave **6** in 98% HPLC conversion and 95% purity (entry 6) and thus became the method of choice (Scheme 2, step 4). Reducing the amount of TEMPO by half resulted in only ca. 80% conversion (see the SI, Table S10). The potential for recycling the reaction medium was also demonstrated, further decreasing the environmental footprint for this key oxidation. That is, following the initial reaction and extraction of the aqueous phase that contains catalyst/TBAB/KBr, a second oxidation was carried out by adding recovered toluene and **11**, resulting in 79% conversion. However, an additional 5 mol % TEMPO and 0.25 equiv of NaClO increased the conversion to 90% (see the SI, S19).

## Conclusions

A straightforward and potentially cost-effective synthesis of ketone **6** has been developed, which is a key component in the synthesis of the potent anti-HIV drug lenacapavir. The approach features a 1,2-addition to an aldehyde that relies on an in situ-generated organozinc halide complex from the commercially available Grignard reagent difluorobenzylmagnesium bromide. Oxidation of the newly formed secondary alcohol **11** can be effectively carried out using catalytic amounts of TEMPO in the presence of NaClO as terminal oxidant. This four-step sequence leads, in 67% overall yield, to the targeted intermediate **6**, a route that has significant potential for scale-up.

## Data Availability

The data underlying this study are available in the published article and its online Supporting Information.

## Supporting Information Available

Experimental procedures, optimization details, and analytical data (NMR, HPLC, and MS) (PDF)
