# Supplementary material for: Preparation of a Key Intermediate En Route to the Anti-HIV Drug Lenacapavir
Source: J Org Chem. 2024 Mar 6;89(6):3995–4000. doi: 10.1021/acs.joc.3c02855 (PMC10949239; doi:10.1021/acs.joc.3c02855)
Supplement: Supplementary file 1 — jo3c02855_si_001.pdf [file jo3c02855_si_001.pdf]

Supporting Information

Preparation of a key intermediate *en route* to the anti-HIV drug lenacapavir

Juan C. Caravez, Yuting Hu, Erfan Oftadeh, Kirubel T. Mamo, Bruce H. Lipshutz*

Department of Chemistry and Biochemistry, University of California, Santa Barbara, CA 93106 USA

Phone: 805-893-2521
Fax: 805-893-8265
Email: lipshutz@chem.ucsb.edu
Website: https://lipshutz.chem.ucsb.edu/

Table of Contents

1. General information **S2**
2. Synthetic Schemes **S4**
   Scheme S1: Retrosynthesis of ketone 6 ..... S4
   Scheme S2: Overall synthesis S4
3. Experimental section **S5**
   Table S1. Optimization of step 1 ..... S5
   Table S2. Optimization of step 2 ..... S6
   Scheme S3. One-pot synthesis of aldehyde 3a S7
   Table S3. Optimization of step 3 ..... S9
      Procedure using $ZnBr_2$: S9
      Procedure using $ZnCl_2$: S10
   Table S4. Initial optimization of step 4 using various oxidation conditions S11
   Optimization of step 4 using a nitroxyl radical catalyst S13
   General procedure for oxidation of 5a to 6a using TEMPO as catalyst (1.5 mmol): S13
   Table S5. Preliminary screening of different oxidizing agents S15

Table S6. Preliminary screening of NaClO loading in DCM as the organic solvent S15
Table S7. Solvent screening S16
Table S8. Screening of NaClO equivalents in Toluene as the organic solvent S16
Table S9. Correlation of HPLC purity and practical yield S17
Table S10. Screening of nitroxyl radical catalyst S17
Recycle Study S19
Synthesis of Weinreb amide 2b S20
Table S11. Synthesis of Weinreb amide S20
4. Cost comparisons between oxidation catalyst **S21**
Table S12. Cost analysis of catalyst for oxidation S21
5. HPLC data **S22**
6. References **S28**
7. Analytical data **S29**
8. NMR Spectra **S32**

1. General information

Reagents:

Reagents were purchased from Sigma-Aldrich, Reike Metals, Combi-Blocks and were used without further purification unless noted otherwise.

Note: Although the concentration of 3,5-difluorobenzylmagnesium bromide is predetermined by the vendor, it is highly recommended that the Grignard solution be titrated prior to use to determine its exact concentration.

Chromatography:

Silica gel TLC plates (UV 254 indicator, thickness 200 mm standard grade, glass backed and 230-400 mesh from Merck) were used. The developed TLC plate was analyzed by a UV lamp (254 nm). The plates were further analyzed with the use of an aqueous ceric ammonium molybdate stain or ethanolic vanillin and developed with a heat gun. Flash chromatography was performed using Silicycle Silicaflash® P60 unbonded grade silica.

NMR:
^{1}H, ^{13}C, and ^{19}F NMR were recorded at 25 °C on either an Agilent Technologies 400 MHz, a Bruker Avance III HD 400 MHz, a Bruker Avance III HD 500 MHz or a Varian Unity Inova 600 MHz spectrometer in D_2O, DMSO-*d*$_6$, CD_3OD, or CD_3CN with residual HOD (^{1}H = 4.79 ppm),

DMSO (^{1}H = 2.54 ppm, ^{13}C = 40.45 ppm), CH_3OH (^{1}H = 3.31 ppm, ^{13}C = 49.15 ppm), or CH_3CN (^{1}H = 1.94 ppm, ^{13}C = 1.39 ppm) as internal standard. Chemical shifts are reported in parts per million (ppm). The data presented will be reported as follows; chemical shift, multiplicity (s = singlet, bs = broad singlet, d = doublet, dd = doublet of doublet, t = triplet, q = quartet, quin = quintet, m = multiplet), coupling constant (if applicable), and integration.

HPLC:

HPLC-grade solvents were obtained from Fisher Scientific. HPLC analysis was performed on an Agilent 1260 series HPLC with a Kromasil 100-5-C18 4.6×50 mm column at the flow-rate of 1mL/min using gradient solvent starting from 40% v/v acetonitrile/water to 98% v/v acetonitrile/water over the course of 12-15 min.

2. Synthetic Schemes

Scheme S1: Retrosynthesis of ketone **6**

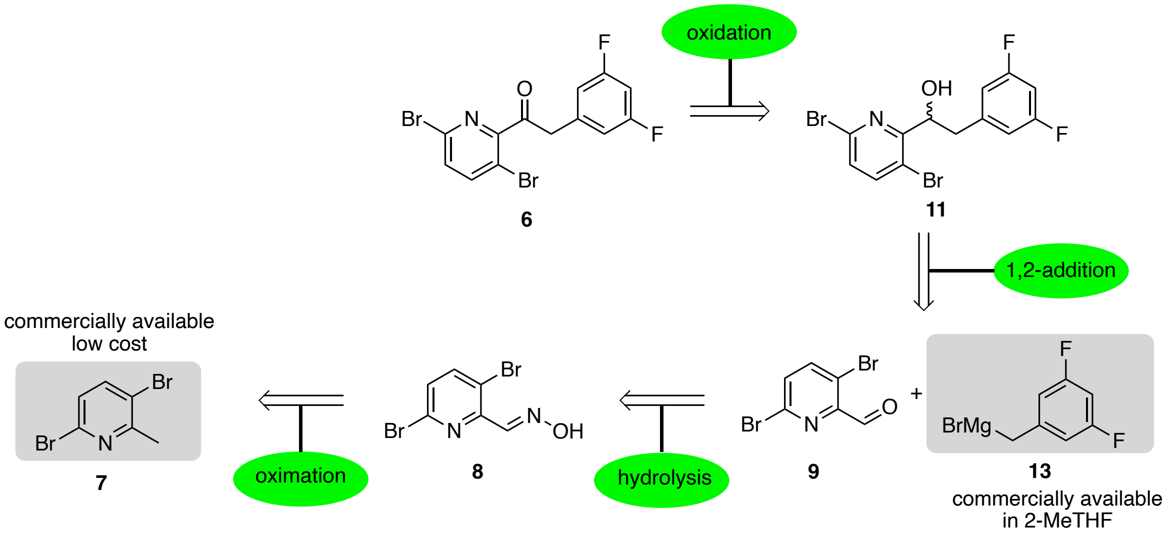

Scheme S2: Overall synthesis

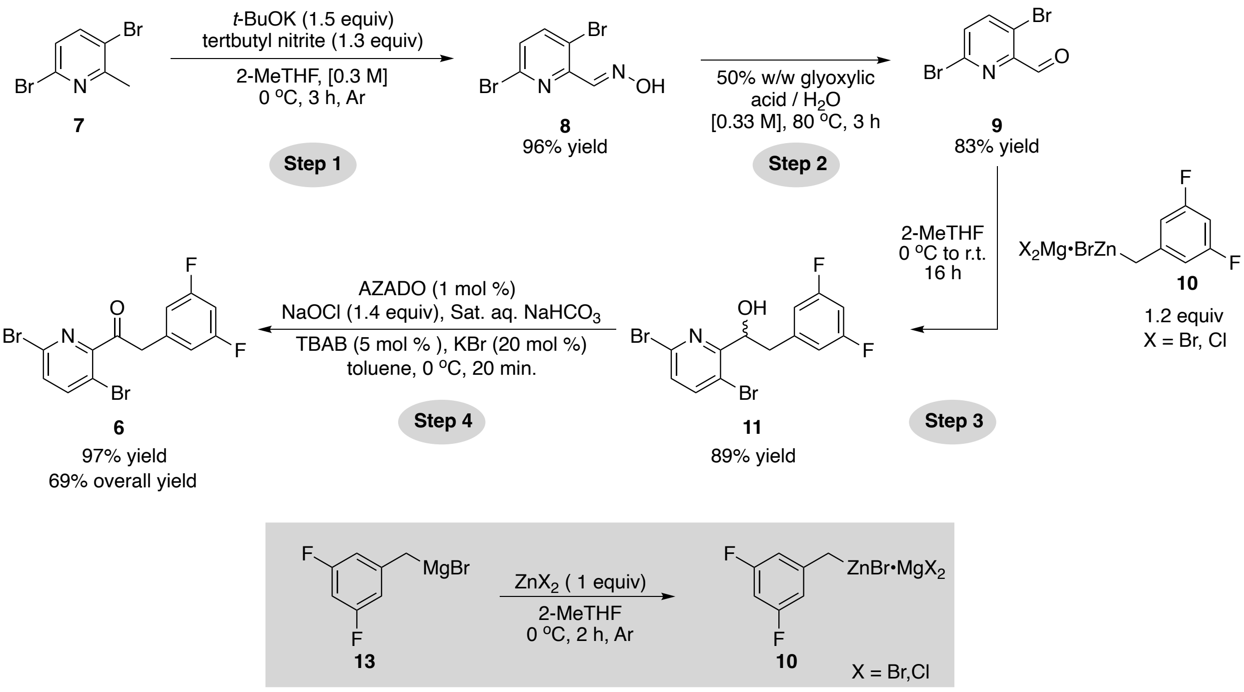

3. Experimental section

Table S1. Optimization of step 1

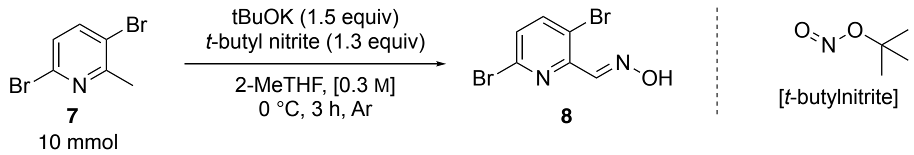

Entry	Equiv of KO*t*Bu	Addition order	Temperature	Solvent	Yield[a]
1	1.0	KO*t*Bu, then *t*-butylnitrite	0 °C to rt	THF	NR
2	1.5	KO*t*Bu, then *t*-butylnitrite	0 °C to rt	THF	11%
3	1.5	*t*-butylnitrite, then KO*t*Bu dropwise	0 °C to rt	THF	45%
4	1.5	*t*-butylnitrite, then KO*t*Bu dropwise	keep at 0 °C	THF	93%
**5**	**1.5**	***t*-butylnitrite, then KO*t*Bu dropwise**	**keep at 0 °C**	2-MeTHF	**94%**[b]
**6**	**1.5**	***t*-butylnitrite, then KO*t*Bu dropwise**	**keep at 0 °C**	2-MeTHF	**96%**[c]

a) Isolated yield. b) Run on a 40 mmol scale. c) Run on a 20 mmol scale.

Synthesis of aldehyde **9** from dibromopicoline **7** has been adapted from literature known procedure[1]

Procedure for a 20 mmol scale reaction:

To an oven dried 250 mL round bottom flask (RBF) equipped with a large magnetic stir was added 2,5-dibromo-6-methylpyridine **7** (5.0184 g, 20 mmol, 1 equiv), then capped with a rubber septum. The RB was then filled with argon and evacuated (3x). Anhydrous 2-MeTHF (50 mL) was then added via syringe while the flask was maintained under a stream of argon. The solution was then cooled to 0 °C followed by the addition of *t*-butyl nitrite (2.68 g, 26 mmol, 1.3 equiv, 3.1 mL) by syringe. A solution of potassium *t*-butoxide in anhydrous 2-MeTHF (1.74 M, 17.24 mL, 30 mmol *t*-BuOK) was added dropwise into the RBF while stirring and maintaining the temperature at 0 °C. The reaction was then stirred at 0 °C for 3 h. After 3 h, the reaction was diluted with 2-MeTHF (15 mL) and quenched with sat. NH_4Cl aqueous solution (25 mL). The 2-MeTHF was then removed *in vacuo* with an 89% recovery of 2-MeTHF. Once all the 2-MeTHF was removed a light-yellow precipitate was left behind in the aqueous phase. The light-yellow solid was then filtered and the filter cake was washed with water (2 x 25 mL) and allowed to dry overnight to afford a light yellow solid (96% yield, 5.347 g, 87% purity by ^{1}H NMR). Oxime **8** was used directly in the next step without further purification.

Note: It is important to use a large enough RBF and stir bar to ensure proper stirring as the product begins to precipitate out shortly after the addition of t-BuOK.

Preparation of *t*-BuOK solution in 2-MeTHF:

Inside an argon filled glove-box, to a 20 mL oven dried volumetric flask was added 3.905 g of *t*-BuOK. The flask was capped with a septum and removed from the glove-box. To the flask was then added anhydrous 2-MeTHF to the volumetric line. To assure complete dissolution the volumetric flask was sonicated for a period of 5 min.

Figure S1. Synthesis of **8**

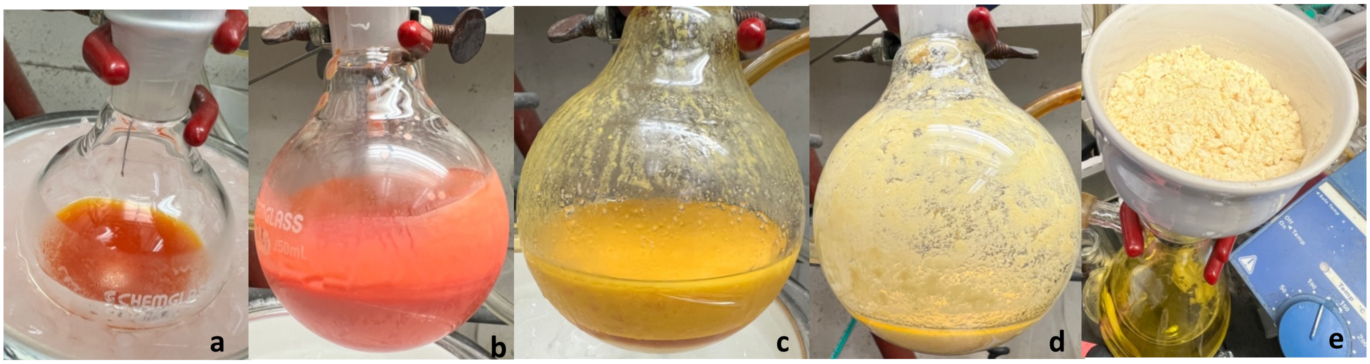

a) Dropwise addition of a solution of *t*-BuOK in 2-MeTHF at 0 ºC. b) Reaction mixture at 3 h. c) Reaction mixture after quenching with Sat. aqueous NH_4Cl. d) Reaction mixture after removing 2-MeTHF. e) Filtration of product.

Table S2. Optimization of step 2

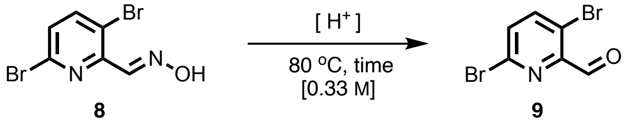

1 mmol

Entry	H^+ source	time	Yield[a]
1	aq. 1 M HCl	3 h	nr
2	Glacial acetic acid: H_2O; 1:1	3 h	nr
3	Levulinic acid neat, 10 v/v %1 M aq HCl	3 h	trace
4	50% w/w glyoxylic acid/H_2O	1 h	29%
5	50% w/w glyoxylic acid/H_2O	2 h	62%
6	50% w/w glyoxylic acid/H_2O	3 h	80%[b]
7	50% w/w glyoxylic acid/H_2O	3 h	86%[c]

a) Isolated yield. b) Run on a 17.5 mmol scale c) run on a 30 mmol scale reaction.

General procedure for hydrolysis of 8

To an appropriately sized flask equipped with a magnetic stir bar was added the acidic reaction medium warmed to 80 ºC using an oil bath for a period of ca. 10 min. Oxime **8** was then added in one portion. The heterogenous mixture was then stirred un a water jacketed reflux condenser for a period of 3 h.

Procedure for a 17.5 mmol scale reaction under optimized conditions:

To 250 mL RBF equipped with a large magnetic stir bar was added a 50% w/w solution of glyoxylic acid in H_2O (48.5 mL). The solution was warmed to 80 °C using an oil bath for ca. 10 min. To the warm solution was then added crude oxime **8** (17.5 mmol, 4.89 g) in one portion via an addition funnel. The yellow suspension was stirred vigorously at 80 °C using an oil bath under a water jacketed condenser for a period of 3 h. The suspension was then cooled to rt and then to 0 °C. The precipitate was vacuum filtered and washed with cold water (25 mL x 3) and dried overnight *in vacuo* to afford a light yellow/golden crystalline solid **9** (3.712 g, 80% yield, 99% pure by ^{1}H NMR). This material was used in the next step without any further purification. *The yield represented in Scheme S2 is the average of entries 6 and 7 in Table S2.*

Figure S2. Synthesis of **9**

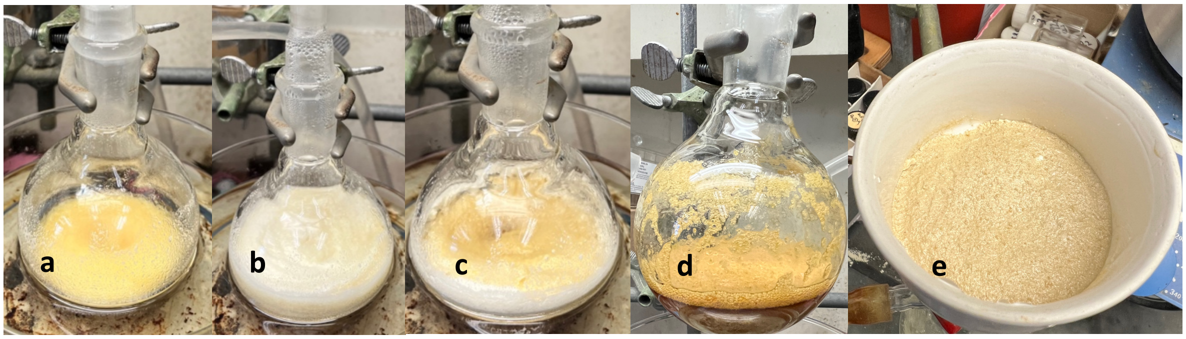

a) After addition of Oxime (2) from previous strep to a solution of 50% w/w glyoxylic acid / H_2O. b) 1 h into reaction. c) 2 h into reaction. d) At 3 h. e) Filtration of aldehyde **9**.

Scheme S3. One-pot synthesis of aldehyde 9

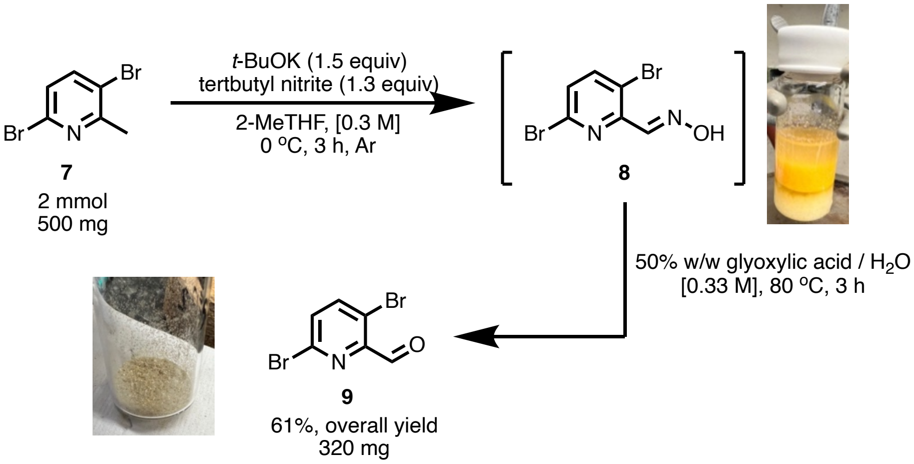

Procedure for one-pot synthesis of aldehyde 9

To an oven dried 6-dram vial equipped with large magnetic stir bar was added **7** (500 mg, 2 mmol). The vial was capped with a septum, filled with argon and evacuated 3 times. Under a stream of argon was added anhydrous 2-MeTHF (5 mL) followed by the addition of *t*-butylnitrite (1.3 equiv, 2.6 mmol, 309 μL). The solution was cooled to 0 ºC in an ice bath and from a previously made stock solution in 2-MeTHF (1.76 M) (see procedure for step 1, above) *t*-BuOK (1.5 equiv, 1.74 M, 1.72 mL) was added dropwise. The reaction mixture was stirred at 0 ºC using an ice bath for 3 h. The yellow heterogeneous mix was diluted with 2-MeTHF (3 mL) and quenched with sat. aq NH_4Cl (5 mL). The 2-MeTHF was removed by rotary evaporation to afford a yellow precipitate in the aqueous layer. The vial was centrifuged for a period of 2 min. The supernatant was removed by syringe. To the vial was then added water (1.5 mL), centrifuged and the water was removed by syringe. This process was repeated 5 times to afford a yellow residue, crude **8**.

To the same vial containing the crude material from the last step was added water (2 mL) and warmed to 80 ºC using an aliminum heating block for a period of ca. 5 min. To the warm mixture was added a 50% w/w solution of glyoxylic acid in water (5.6 mL) dropwise via syringe. The mixture was then stirred at 80 ºC for a period of 3h. The reaction mixture was then cooled to rt then to 0 ºC. The heterogeneous mixture was centrifuged and the remaining acidic solution was removed by syringe. To the vial was then added water (1.5 mL), stirred, and centrifuged following removal of the supernatant. The process was repeated 4 more times. The light yellow/golden material was then dried at 40 ºC using an aluminum heating block overnight to afford aldehyde **9** (320 mg, 61% yield).

Note: Due to the small scale associated with this two step one-pot sequence it is suspected that some material was lost during the aqueous washes at both steps. However, this demonstrates the potential to carry out an efficient one-pot sequence to synthesize aldehyde ***9*** *from the corresponding dibromopicoline 7 when run at a larger scaled using the appropriate reactors.*

Table S3. Optimization of step 3

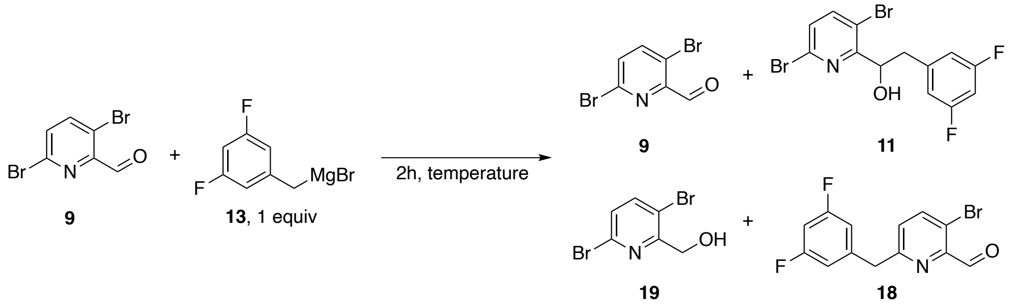

Entry[a]	Temperature	difference from above	9 (%)[d]	11 (%)[d]	18 (%)[d]	19 (%)[d]
1	0 °C to rt	-	11	34	44	3
2	keep at 0 °C	-	9	38	40	6
3	keep at 0 °C	aldehyde : Grignard = 1:1.2	5	14	66	3
4	keep at 0 °C	aldehyde : Grignard = 1:0.8	19	51	23	2
5	keep at 0 °C	aldehyde : Grignard = 1:0.6	35	45	10	2
6	keep at 0 °C	add Grignard dropwise over 2h	42	10	33	5
7	keep at 0 °C	add 1 equiv BF_3OEt_2	44	27	20	6
8	keep at 0 °C	add aldehyde dropwise into Grignard reagent	3	35	53	2
9	keep at 0 °C	mix 1 equiv CuCN with Grignard, then use 0.8 equiv	12	43	36	6
10	keep at 0 °C	use organozinc reagent made from bromide and zinc dust	11	60	27	nd
11	keep at 0 °C	mix 1 equiv $ZnBr_2$ with Grignard, then use 0.8 equiv mixture	~50 % SM left with 2h			
12	0 °C to rt	mix 1 equiv $ZnBr_2$ with Grignard, then use 0.8 equiv mixture, 12 h	20	62	4	2
13	0 °C to rt	mix 1 equiv $ZnBr_2$ with Grignard, then use 1.0 equiv mixture, 12 h	15	80	3	2
**14**	**0 °C to rt**	**mix 1 equiv $ZnBr_2$ with Grignard, then use 1.2 equiv mixture, 12 h**	**8**	**88**	**2**	**1**
15	0 °C to rt	mix 1 equiv $ZnCl_2$ with Grignard, then use 1.3 equiv mixture, 20 h	—	73[c]	—	—
**16[e]**	**0 °C to rt**	**mix 1 equiv $ZnCl_2$ with Grignard, then add solution of 9 dropwise 16 h**	—	**89[c]**	—	—

a) Reactions run on a 0.2 mmol scale. b) Run on a 1.5 mmol scale. c) Isolated yield d) Yield determined by GC-MS using naphthalene as internal std.e) Reaction was run on a 0.25 mmol scale.

Procedure using $ZnBr_2$:

To an oven-dried 6-dram vial equipped with a magnetic stir bar was added $ZnBr_2$ (0.24 mmol, 54 mg, 1.2 equiv) in an argon filled glove box. The vial was sealed with a rubber septum, then removed from the glove box. To the vial containing $ZnBr_2$, a Grignard solution of **13** in 2-MeTHF (0.25 M, 1.2 equiv, 0.96 mL) was added. The solution was stirred at 0 °C using ice bath for 2 h or until complete consumption of $ZnBr_2$ was observed. In a separate oven-dried 2-dram vial equipped with a magnetic stir bar was added 3,6-dibromopicolinaldehyde **9** (1 equiv, 0.2 mmol, 53 mg). The vial was sealed with a rubber septum and filled with argon and evacuated three times. Under a stream of argon 50 µL of anhydrous 2-MeTHF was added to the vial. This solution was then cooled to 0 °C in an ice bath and the $ZnBr_2$ / Grignard solution was added dropwise. The vial was then removed from the ice bath and stirred at 23 0 °C for a period of at least 12 h. Upon completion the reaction was cooled to 0 °C then quenched with saturated aqueous NH_4Cl solution (1 mL). The organic layer was then separated and the aqueous layer was extracted with diethyl ether (1 mL x

4). The organic layers were combined, dried, concentrated *in vacuo*. The crude material was then subjected to quantitative GC-MS analysis using naphthalene as an internal standard to determine a yield of 88%.

Procedure using $ZnCl_2$:

To an oven-dried 6-dram vial equipped with a magnetic stir bar was added $ZnCl_2$ (1.3 equiv, 0.33 mmol, 44.3 mg) in an argon filled glove box. The vial was sealed with rubber septum then removed from the glove box. To the vial containing $ZnCl_2$, a Grignard solution of **13** in 2-MeTHF (0.25 M, 1.3 equiv, 1 mL) was added. The solution was stirred at 0 °C for 2 h or until complete consumption of $ZnCl_2$ was observed. In a separate oven dried 2-dram vial equipped with a magnetic stir bar was added 3,6-dibromopicolinaldehyde **9** (1 equiv, 66.2 mg). The vial was sealed with a rubber septum and filled with argon and evacuated three times. Under a stream of argon 0.5 mL of anhydrous 2-MeTHF was added to the vial. The 6-dram vial containing the $ZnCl_2$ Grignard solution was cooled to 0 °C and the solution of 2-MeTHF containing **9** was added via syringe dropwise while under a stream of argon. Anhydrous 2-MeTHF (0.5 mL) was then added to the vial in which the 3,6-dibromo-picolinaldehyde **9** and then transferred to the reaction vial containing the $ZnCl_2$ / Grignard solution. The vial was removed from the ice bath and stirred at 23 0 °C for a period of 16 h. Upon completion the reaction was cooled to 0 °C then quenched with saturated aqueous NH_4Cl solution (1 mL). The organic layer was then separated and the aqueous layer was extracted with diethyl ether (4 x 1 mL). The organic layers were combined, dried, concentrated *in vacuo* and purified by column chromatography using a gradient of 10-15% EtOAc/hexanes to afford the alcohol **5a** as a white solid (87 mg, 89% yield).

Table S4. Initial optimization of step 4 using various oxidation conditions

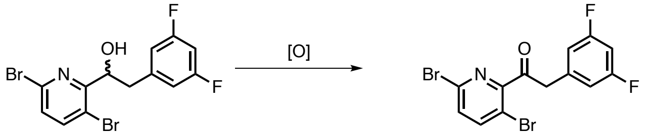

11 6

Entry[c]	conditions	Temperature	Conversion %	Yield %[b]
1[a]	1.5 equiv Dess-Martin periodinane, 2 wt % TPGS-750-M/H_2O, 24 h	45 °C	trace	<5
2[a]	1.5 equiv Dess-Martin periodinane, THF, 24 h	45 °C	92	65(63)[f]
3	5 mol % CuBr, 5 mol % TEMPO, 5 mol % bipyridine, 10 mol % NMI 2 wt TPGS-750-M/H_2O, 24 h, open vial	70 °C	7	nd
4	25 mol % $Cu(OAc)_2$•H_2O , 2 equiv NH_4NO_3, MeCN:H_2O, 24 h	70 °C	0	nd
5	30 mol % **20**, 1.0 equiv Oxone, MeCN: H_2O, 6 h	70 °C	0	nd
6	2 equiv of **20,** EtOAc, 6 h	70 °C	0	nd
7	10 mol % SnCl2, 30 mol % KBr, 5 equiv H2O2, dioxane, 24 h	rt	10	5
8	10 mol % AlMe3, 3.0 equiv m-Nitro-benzaldehyde, toluene, 3 h	0 °C to rt	0	nd
9[d]	1 mol % **16**, CH_3CN (0.25 M), Oxone (0.6 equiv), opent to air, 24 h	70 °C	30	24
10[d]	5 mol % **16**, CH_3CN (0.25 M), Oxone (1.2 equiv), open to air, 24 h	70 °C	–	65[f]
11[d]	10 mol % **16**, CH_3CN (0.25 M), Oxone (1.2 equiv), open to air, 24 h	70 °C	–	75[f]
12[e]	10 mol % **16**, CH_3CN (0.25 M), Oxone (1.2 equiv), open to air, 24 h	70 °C	–	79[f]
**13[d]**	**10 mol % 16, CH_3CN (0.25 M), Oxone (2.4 equiv), open to air, 24 h**	**70 °C**	**–**	**90[f]**

a) Run on a 0.1 mmol scale. b) Yield determined by qNMR using 1,3,5-trimethoxybenzene as IS. c) Run on 0.2 mmol scale. d) run on a 0.25 mmol scale. e) Run on a 1 mmol scale. f) Isolated yield.

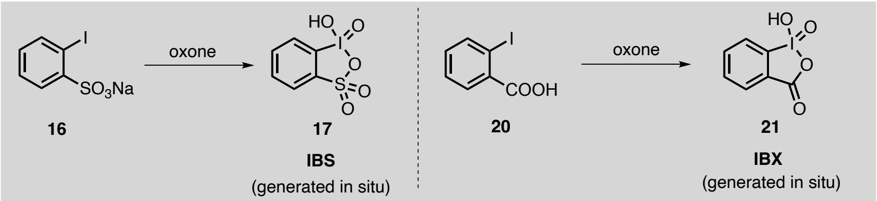

Procedure for entries 1 and 2[2]:

To a 1-dram vial equipped with a magnetic stirrer, 1-(3,6-dibromopyridin-2-yl)-2-(3,5-difluorophenyl)ethan-1-ol, **11** (39.3 mg, 0.10 mmol, 1 equiv), Dess-Martin periodinane (63.6 mg, 0.15 mmol, 1.5 equiv) and the corresponding solvent (0.2 mL) were added. The reaction vial was capped with a screw cap. The mixture was stirred vigorously at 45° C. After the indicating time, the reaction was monitored by TLC. For reactions in 2 wt % aqueous TPGS-750-M, the reaction was extracted with EtOAc (2 mL x 3). Then the yield was obtained by qNMR (1,3,5-trimethoxybenzene as internal standard). For reactions in THF, the reaction was filtered with a short silica plug to remove the inorganic salt. Then the yield was obtained by ^{1}H qNMR (1,3,5-trimethoxybenzene as internal standard), resulting in 92% conversion and 65% yield. The isolated yield for entry 2 was 63%.

Procedure for entry 3[3]

To a 1-dram vial equipped with stir bar was added the 1-(3,6-dibromopyridin-2-yl)-2-(3,5-difluorophenyl)ethan-1-ol, **11** (78.6 mg, 0.20 mmol, 1 equiv), followed by CuBr (1.5 mg, 0.01 mmol, 0.05 equiv), bipyridine (1.6 mg, 0.01 mmol, 0.05 equiv), TEMPO (1.6 mg, 0.01 mmol, 0.05 equiv), NMI (1.6 µL, 1.6 mg, 0.02 mmol, 0.1 equiv) and 2 wt % TPGS-750-M/H_2O solution (0.4 mL). The reaction was vigorously stirred at rt for 24 h with exposure to air. The reaction was

extracted with EtOAc (2 mL x 3). The yield was obtained by qNMR (1,3,5-trimethoxybenzene as internal standard), resulting in 7% conversion; no product detected.

Procedure for entry 4:

To a 1-dram vial equipped with stir bar was added 1-(3,6-dibromopyridin-2-yl)-2-(3,5-difluorophenyl)ethan-1-ol, **11** (78.6 mg, 0.20 mmol, 1 equiv), followed by $Cu(OAc)_2{\cdot}H_2O$ (10 mg, 0.05 mmol, 0.25 equiv), NH_4NO_3 (32 mg, 0.4 mmol, 2 equiv), acetonitrile (0.1 mL) and water (0.1 mL). The reaction was vigorously stirred under 70 °C for 24 h. The reaction was cooled to rt and then in an ice bath. Then the mixture was filtered. The solid was collected dissolved in $CDCl_3$. The yield was obtained by qNMR (1,3,5-trimethoxybenzene as internal standard), resulting in 0% conversion; no product detected.

Procedure for entry 5[4]:

To a 1-dram vial equipped with stir bar was added 1-(3,6-dibromopyridin-2-yl)-2-(3,5-difluorophenyl)ethan-1-ol, **11** (78.6 mg, 0.20 mmol, 1 equiv), 2-iodobenzoic acid (15 mg, 0.06 mmol, 0.3 equiv), Oxone (69.2 mg, 0.2 mmol, 1.0 equiv), acetonitrile (1.7 mL) and H_2O (0.88 mL). The reaction was vigorously stirred at 70 °C for 6 h. The reaction was cooled to rt and then filtered with a short silica plug to remove the inorganic salt. The yield was obtained by qNMR (1,3,5-trimethoxybenzene as internal standard), resulting in 0% conversion; no product detected.

Procedure for entry 6:

To a 1-dram vial equipped with stir bar was added the 1-(3,6-dibromopyridin-2-yl)-2-(3,5-difluorophenyl)ethan-1-ol, **11** (78.6 mg, 0.20 mmol, 1 equiv), followed by 2-iodobenzoic acid (99 mg, 0.4 mmol, 2.0 equiv) and EtOAc (0.5 mL) . The reaction was vigorously stirred at 70 °C for 24 h. The reaction was filtered with a short silica plug to remove the inorganic salt. Then the yield was obtained by qNMR (1,3,5-trimethoxybenzene as internal standard), resulting in 0% conversion; no product detected.

Procedure for entry 7[5]:

To a 1-dram vial equipped with stir bar was added the 1-(3,6-dibromopyridin-2-yl)-2-(3,5-difluorophenyl)ethan-1-ol, **11** (78.6 mg, 0.20 mmol, 1 equiv), followed by $SnCl_2{\cdot}2H_2O$ (4.5 mg, 0.02 mmol, 0.1 equiv), KBr (7.1 mg, 0.06 mmol, 0.3 equiv) and dioxane (0.2 mL). The reaction was stirred under rt. H_2O_2 (30 % w/w, eq, 0.1 mL, 1 mmol, 5 equiv) were added by 5 portions over 2 h (1 portion every 30 min). The reaction was stirred at rt for 24 h. Then the reaction was filtered with a short silica plug to remove the inorganic salt. Then the yield was obtained by qNMR (1,3,5-trimethoxybenzene as internal standard), resulting in 10% conversion; 5% product was detected.

Procedure for entry 8[6]:

To a 1-dram vial equipped with a stir bar was added 1-(3,6-dibromopyridin-2-yl)-2-(3,5-difluorophenyl)ethan-1-ol, **11** (78.6 mg, 0.20 mmol, 1 equiv). Then the vial was flushed with argon three times. Anhydrous toluene (0.63 mL) was added by syringe, followed by $AlMe_3$ (10 μL of a

2 M solution in toluene, 0.02 mmol) via syringe. The reaction was stirred at 0 °C for 10 min after which 3-nitrobenzaldehyde (110.5 mg, 0.6 mmol) in anhydrous toluene (0.167 mL) was added and the reaction was stirred at rt for 3 h. After 3 h, the reaction was diluted with CH_2Cl_2 (3 mL) and washed with 1 M aqueous HCl (1.6 mL), saturated aqueous $NaHCO_3$ (1.6 mL), and H_2O (1.6 mL). The organic layer was collected, dried over anhydrous Na_2SO_4, and concentrated under vacuum. Then the yield was obtained by qNMR (1,3,5-trimethoxybenzene as internal standard), resulting in no conversion; no product detected.

Procedure for entry 13[7]:

To a thick-walled test tube equipped with a magnetic stir was added 1-(3,6-dibromopyridin-2-yl)-2-(3,5-difluorophenyl)ethan-1-ol, **11** (1 equiv, 0.25 mmol, 98 mg), catalytic amounts of **16** (10 mol %, 7.65 mg), powdered Oxone,® potassium peroxymonosulfate (MW: 307.38; 2.4 equiv, 6 mmol, 184.4 mg), followed by the addition of acetonitrile (0.5 mL). The heterogeneous mixture was stirred at 70 °C for 24 h under a water-jacketed condenser. The heterogenous mixture was then cooled to rt and the acetonitrile was removed by rot-evap. The reaction mixture was re-dissolved in EtOAc (ca. 1.5 mL) and washed with water (1.5 mL x 3). The aqueous layer was extracted with EtOAc (1 mL x 2). The organics were combined and concentrated *in vacuo*. The crude residue was dissolved in minimal EtOAc and passed through shorth silica plug (1/3" x 4") using a gradient of 10-20% EtOAc/hexanes to afford a white solid (88.4 mg, 90% yield, 96% purity by HPLC).

Note: Oxone was ground with a mortar and pestle into a fine powder prior to use.[7] Optimization towards the oxidation of alcohol ***11*** *to ketone* ***6*** *was done in parallel with the use of a nitrosyl-like catalyst. Although this method proceeds to be efficient for the oxidation, the focus was turned to using a potentially more cost-effective set of conditions (NaOCl and a nitrosyl-like catalyst).*

Optimization of step 4 using a nitroxyl radical catalyst

General procedure for oxidation of **11** to **6** using TEMPO as catalyst[8] (1.5 mmol):

To a 100 mL RBF equipped with a magnetic stir bar, 1-(3,6-dibromopyridin-2-yl)-2-(3,5-difluorophenyl)ethan-1-ol, **11** (589.5 mg, 1.5 mmol), TEMPO (23.4 mg, 10 mol%), TBAB (24.15 mg, 5 mol %) and KBr (35.7 mg, 20 mol %) were added followed by addition of saturated aq. solution of $NaHCO_3$ (1.8 mL) and then, toluene (7.5 mL, 0.2 M). The reaction mixture was then pre-mixed for 5 min at 0 °C. While maintaining the reaction mixture at 0 °C, a premixed cooled solution (0 °C) of 10-15% aq. NaOCl: sat. aq $NaHCO_3$ (1:1.4, 1.21 mL:1.7 mL : 2.9 mL) was added to the reaction flask at which it was stirred for 10 min. HPLC analysis of the corresponding reaction showed the presence of **11** (10%) in the crude reaction mixture. Accordingly, an additional NaOCl (0.1 equiv, 0.092 mL) was added and the reaction was stirred for another 10 min. Another HPLC sample was prepared and the analysis indicated 97% conversion of **11** to **6**. To avoid product decomposition (over-oxidation), no further NaOCl was added and the reaction was quenched with sat. $Na_2S_2O_3$ (1 mL), after which the crude reaction mixture was extracted with EtOAc (5 mL x 3). The organics were combined and concentrated in vacuo, and then subjected to qNMR and HPLC analysis (90% yield, 91% purity). For further purification, the crude product was subjected to recrystallization.

Figure S3. Oxidation of 5a using TEMPO followed by recrystallization

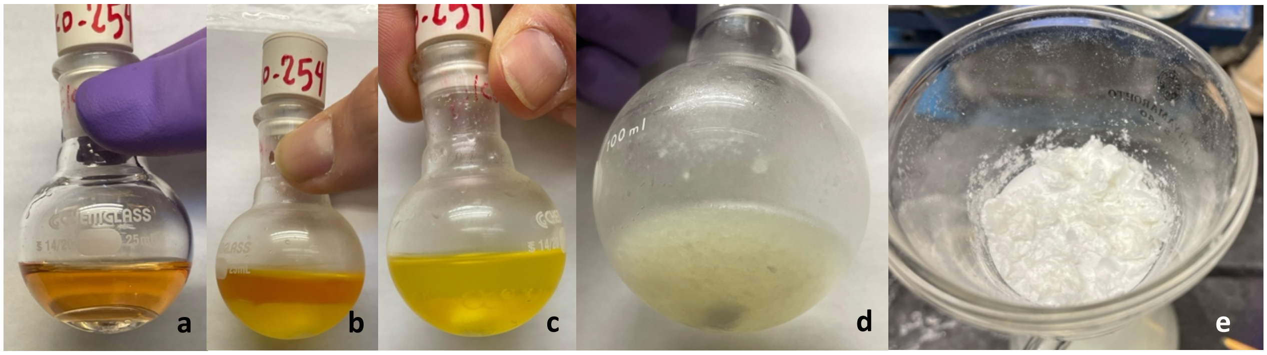

a) Reaction mixture prior to adding NaOCl. b) 10 min. into reaction. c) 20 min. into reaction. d) Recrystallization of **6a**. e) Filtration of crystals.

Recrystallization procedure for 6:

To a 100 mL RBF equipped with a magnetic stir bar, the collected crude sample of **6** (585 mg) and acetonitrile (11.70 mL, 20 volume/g of a sample) was added while mixing (medium speed) at rt. Once a clear solution was observed, DI water (2.92 mL, 5 volume/g of a sample) was added to the flask. The solution was stirred for 10 min followed by the addition of another portion of DI water (2.92 mL, 5 volume/g of a sample). Once the tiny crystals had formed, the dispersion was stirred for 1 h at a low stir rate (ca. 200 rpm). After ca. 30 min, another portion of DI water (5.85 mL, 10 volume/g of a sample) was added to the solution and solution was mixed for 30 min. After 30 min, the final portion of DI water (5.85 mL, 10 volume/g of a sample) was added and the dispersion was stirred for another 30 min. The solution was vacuum filtered and washed with DI water (3 x 2 mL) and dried to afford **6** (440 mg, 75% mass recovered) with 98% purity based on HPLC peak area.

Note: Because of its crystalline nature, ***6*** *can easily undergo recrystallization as a preferred method for purification (as described above). Due the small academic scale on which this work was done, the mass recovery of the pure product was ca. 75%.*

Table S5. Preliminary screening of different oxidizing agents

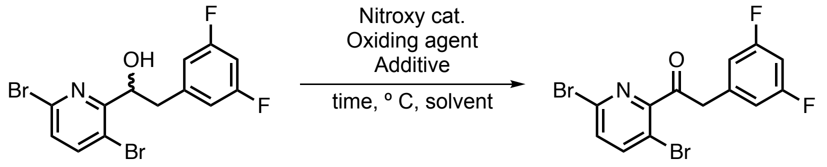

11
0.1 mmol

6

Entry	Nitroxy cat. (Mol %)	Oxdizing Agent	Additive	Temperature	Time	Solvent	Yield (%)[a]
1	TEMPO (5 mol%)	1.1 eq. PIDA	none	rt	1 hr	*t*-Butanol	No rxn
3	Azado (2 mol %)	1.1 eq. PIDA	none	50 ºC	24 hr	AcOH	No rxn
2	Azado (1 mol %)	O_2	$NaNO_2$	rt	24 hr	AcOH	No rxn
4[b]	Azado (17 mol %)	1.5 eq NaOCl	KBr/TBAB/$NaHCO_3$	0 ºC	1 hr	DCM	40
5[c]	Azado (1 mol%)	1.5 eq NaOCl	KBr/TBAB/$NaHCO_3$	0 ºC	8 min	DCM	72

a) 1,3,5-trimethoxybenzene (CAS:621-23-8) was used as an internal standard b) 10 mol% KBr, 5 mol% TBAB and saturated aqueous solution of $NaHCO_3$ was used. c)20 mol% KBr, 5 mol% TBAB and saturated aqueous solution of $NaHCO_3$ was used

Table S6. Preliminary screening of NaClO loading in DCM as the organic solvent

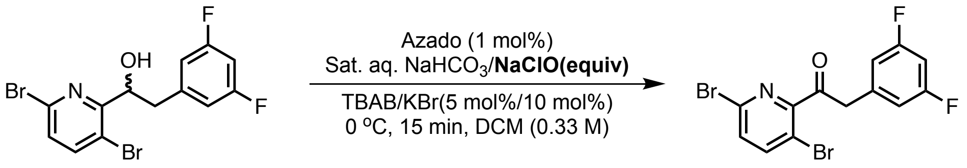

11
0.10 mmol

6

Entry	NaOCl (equiv)	Conversion (5a)[a]	6a (%) [b]	Side Product/s (%)[c]
1	1.0	36	24	12
2	1.1	60	45	15
3	1.2	64	48	16
4	1.3	90	78	11
5	1.4	98	82	16
6[d]	1.4	61	60	1
7	1.5	98	68	30

a) Consumption of 11 based on HPLC area peak b) Conversion of 11 towards 6 based on HPLC area peak c) Conversion of 11 towards side product/s based on HPLC area peak d) The reaction was setup at -10 ºC

Note: Initial screenings were done in DCM to gain insight into the efficiency of this transformation. Once a set of conditions were established, a solvent screening was carried out to find a non-chlorinated and potentially recoverable solvent (see Table S7).

Table S7. Solvent screening

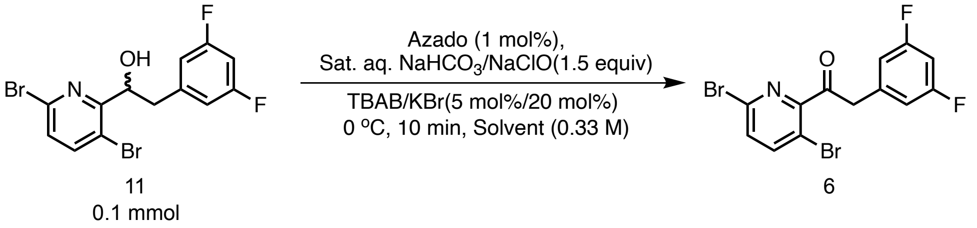

Entry	Solvent	Conversion (5a)[a]	6a (%) [b]	Side Product/s (%)[c]
1	Ethyl Acetate	93	88	5
2	Acetone	22	2	20
3	MTBE	49	1	48
4	MeCN	43	6	37
5	Toulene	78	76	2

a) Consumption of 11 based on HPLC area peak b) Conversion of 11 towards 6 based on HPLC area peak c) Conversion of 11 towards side product/s based on HPLC area peak

Table S8. Screening of NaClO equivalents in Toluene as the organic solvent

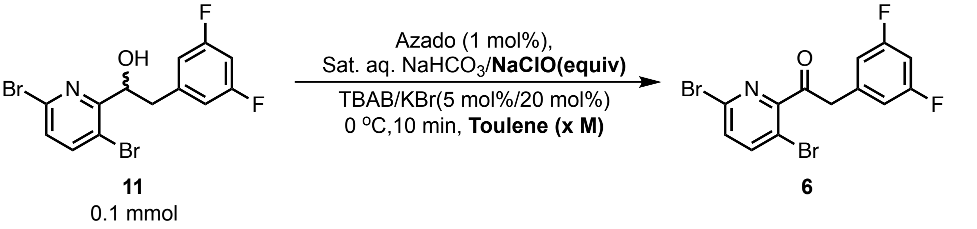

Entry	NaOCl	Concentration	Conversion (11)[a]	6 (%) [b]	Side Product/s (%)[c]
1	1.2	0.33	96	93	3
2	1.2	0.5	98	94	4
3	1.25	0.2	99	96	3
4[d]	1.25	0.2	99	97	2
5	1.2	0.2	99	96	3

a) Consumption of 11 based on HPLC area peak b) Conversion of 11 towards 6 based on HPLC area peak c) Conversion of 11 towards side product/s based on HPLC area peak d) the reaction was performed in 0.25 mmol scale

*Note: NaClO typically comes in a solution with a range of concentration (10-15%). For this study, no titration was done although it is strongly recommended that the accurate concentration of the active chloride be determined prior to usage. It is worth mentioning that knowing this value would help to get highly pure product (**6**) to the extent that no purification might be needed.*

Table S9. Correlation of HPLC purity and practical yield

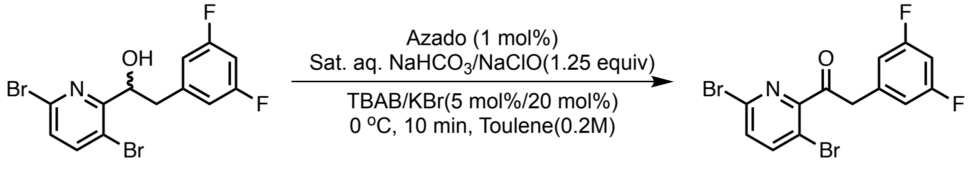

11 0.25 mmol → **6**

HPLC purity (%)[a]	qNMR (%)[b]	Isolated yield (%)
98	97	97

a) Based on the area peak of the 6 (at 210 nm) compared to all the ptresent peaks in the sample (excluding the peak of toluene which was used as a solvent) b) 1,3,5-trimethoxybenzene was used as an internal standard.

Note: qNMR and isolated yield were derived from crude product and results indicated that ***6*** *was formed in 97% yield in both cases. This yield was within 1% deviation from HPLC purity taken initially. This result was an indication that there is a close correlation between HPLC purity and actual yield as seen in the Table above. Consequently, HPLC was conveniently used to monitor the efficiency of all oxidation reactions that were run using N-oxyl catalyst,s unless noted otherwise.*

Table S10. Screening of nitroxyl radical catalyst

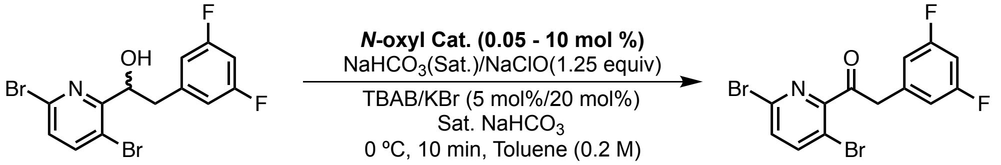

11 0.1 mmol → **6**

Entry	Catalyst	Loading (mol %)	Conversion (**11**)[a]	**6** (%)[b]	Side Product/s (%)[c]
1[d]	AZADO	1	99	97	2
2	AZADO	0.5	99	93	6
3	AZADO	0.25	96	95	1
4	AZADO	0.1	65	58	7
5	AZADO	0.05	30	28	2
6	ABNO	1	99	98	1
7	Keto-ABNO	5	52	32	20
**8**	**TEMPO**	**10**	**98**	**95**	**3**
9	TEMPO	5	80	80	-

a) Consumption of 11 based on HPLC area peak b) Conversion of 11 towards 6 based on HPLC area peak c) Conversion of 11 towards side product/s based on HPLC area peak.
d) Run on a 0.25 mmol scale.

Figure S4. Nitroxyl catalysts

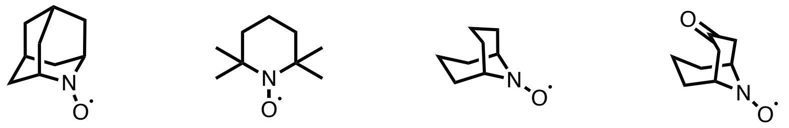

AZADO CAS: 57625-08-8 | **TEMPO** CAS:2564-83-2 | **ABNO** CAS: 31785-68-9 | **KETO-ABNO** CAS: 7123-92-4

Experimental procedure for the use of AZADO (0.25 mmol scale)

To a 1-dram vial equipped with a magnetic stir bar, 1-(3,6-dibromopyridin-2-yl)-2-(3,5-difluorophenyl)ethan-1-ol, **11** (98.7 mg, 0.25 mmol), 0.10 mL of Azado stock solution (1.9 mg dissolved in 0.5 mL of toluene; 1 mol %) and toluene (1.15 mL, 0.2 M) were added. Then, TBAB (4.1 mg, 5 mol %) and KBr (6.0 mg, 20 mol %) were added to the reaction mixture followed by addition of saturated aq. solution of $NaHCO_3$ (0.3 mL). The reaction mixture was pre-mixed for 1 min at 0 ºC and while maintaining the reaction mixture at 0 ºC, a pre-mixed cool solution (0 ºC) of 10-15% aq. NaOCl: sat. aq. $NaHCO_3$ (1:1.4)(0.21 mL:0.294 mL) was added to the reaction vial which was stirred for 10 min at (0 ºC). The crude reaction mixture was extracted with EtOAc (3 x 1 mL) and the organic layers were combined and a small portion (15 µL) was subjected to HPLC analysis (98% HPLC peak area purity). The collected organic phase was concentrated *in vacuo* affording a total mass of 98.9 mg of crude sample. Quantitative NMR analysis of the crude sample gave 97% yield of **6**. The sample was then prepared for recrystallization.

Recycle Study

Figure S5. Recyclability of catalyst

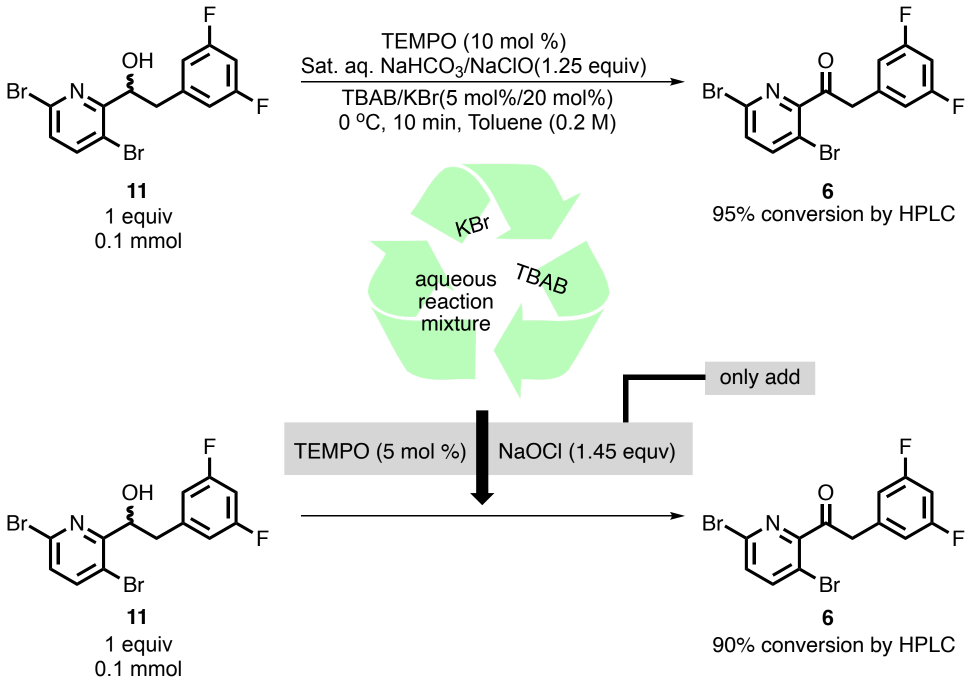

Procedure for recycling of the oxidation reaction medium using NaOCl and TEMPO

Initial reaction

To a 1-dram vial equipped with a magnetic stir bar, 1-(3,6-dibromopyridin-2-yl)-2-(3,5-difluorophenyl)ethan-1-ol, **11** (39.3 mg, 0.1 mmol), TEMPO (1.56 mg, 10 mol %), TBAB (1.66 mg, 5 mol %) , KBr (2.38 mg , 20 mol%) were added followed by addition of saturated aq. solution of $NaHCO_3$ (0.12 mL) and toluene (0.5 mL, 0.2 M). The reaction mixture was then pre-mixed for 5 min at 0 °C. While maintaining the reaction mixture at 0 °C, a pre-mixed cooled solution (0 °C) of 10-15% aq. NaOCl: sat. aq $NaHCO_3$ (1:1.4, 84 µL:117 µL; 0.2 mL) was added to the reaction vial which was stirred for 10 min. HPLC analysis of the corresponding reaction showed 95% conversion to **6**. The aqueous layer was then collected and reused for the next cycle.

First recycle

To a separate 1-dram vial having a magnetic stir bar, 1-(3,6-dibromopyridin-2-yl)-2-(3,5-difluorophenyl)ethan-1-ol, **11** (39.3 mg, 0.1 mmol) and toluene (0.5 mL, 0.2 M) were added. Next, the collected aqueous layer from the previous reaction was transferred into the vial and the whole mixture was stirred at 0 °C for 5 min. Next, a premixed cooled solution (0 °C) of 10-15% aq. NaOCl: sat. aq $NaHCO_3$ (1:1.4, 84 µL:117 µL; 0.2 mL) was added to the reaction mixture slowly and the reaction was stirred for 10 min. The reaction was monitored by HPLC indicating that

another portion of TEMPO (0.78 mg, 5 mol %) and a premixed cooled solution of sat. aq NaOCl : $NaHCO_3$ (1:1.4; 0.03 mL) was required, increasing conversion from 79% to 90%.

Synthesis of Weinreb amide 15

Table S11. Synthesis of Weinreb amide

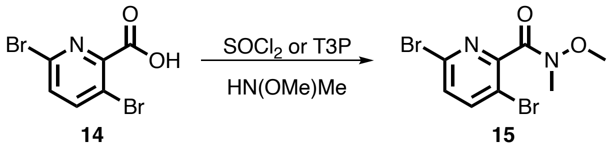

Entry	coupling reagent	time	base	solvent	yield (%)[a]
1[b]	$SOCl_2$	6 h	Et_3N	neat then THF	77%
2[c]	T3P	6 h	DIPEA	EtOAc	83(86)[d,e]

a) Isolated yield. b) Run on a 1.5 mmol scale, c) run on a 5.34 mmol scale. d) Run on a 1 mmol scale, e) Yield by qNMR using 1,3,5-trimethoxybenzene as IS.

Procedure using $SOCl_2$:

Step 1:

To an oven-dried 25 mL RBF equipped with a dry magnetic stir bar was added 3,6-dibromopicolinic acid **14** (1 equiv, 1.5 mmol, 421 mg). The RBF was flushed with argon followed by the addition of $SOCl_2$ (4.5 mL). The mixture was stirred at rt for a 15 min. then at 80 °C for a 3 h. The RBF was then cooled to rt and a reduced pressure distillation apparatus was attached to the RBF. The mixture was warmed to ca. 65 °C and the excess $SOCl_2$ was distilled off. To the RBF was then added anhydrous THF (5 mL) and distilled off to afford the crude acid chloride as a yellow viscous oil.

Step 2:

To an oven-dried 6-dram vial equipped with a dry magnetic stir bar was added *N*,*O*-dimethylhydroxylamine HCl (1.2 equiv, 175.6 mg), dry triethylamine (2 equiv, 3 mmol, 417 µL), and anhydrous THF (3 mL). The suspension was then cooled to 0 °C. Using an oven-dried glass syringe the acid chloride from step 1 was transferred in ca. 1 mL of anhydrous THF and added dropwise. The yellow suspension was stirred at 0 °C to rt for a period of 3 h. Upon completion the dark red reaction mixture was diluted with EtOAc (5 mL), washed with water (2 mL x 2), and the organics were then combined and washed with 1 M NaOH (2 mL), 1 M HCl (2 mL), then brine (2 mL), dried over anhydrous Na_2SO_2, filtered, concentrated *in vacuo* and purified by flash chromatography (10-30% EtOAc/hexanes) to afford and off white solid (375 mg, 77% yield).

Procedure using T3P:

To an oven- dried 50 mL RBF equipped with a magnetic stir bar was added 3,6-dibromopicolinic acid **14** the RBF was then capped with a rubber septum. The RBF was filled with argon and evacuated 3 times. Under a pressure of argon was added anhydrous THF (10.7 mL) and DIPEA (3 equiv, 16.02 mmol, 2.79 mL) the clear faint yellow solution was stirred for 10 min then cooled to -10 °C. Then, dropwise was added a 50% wt solution of T3P in EtOAc (2 equiv, 10.68 mmol, 6.78 mL). The suspension was stirred at -10 °C for 1 h. The reaction mix was warmed to 0 °C, the septum was removed and *N*,*O*-dimethylhydroxylamine HCl (1.5 equiv, 8.01 mmol, 781 mg) was quickly added in 2 portions while maintaining stirring. The mixture was then stirred for 30 min at 0 °C then warmed to rt for 4.5 h monitored by TLC. Upon completion the reaction was diluted with EtOAc (10 mL), quenched with water (8 mL) and then stirred for ca. 10 min. The aqueous was extracted with EtOAc (10 mL x 3), the organics were combined and washed with 1 M HCl (8 mL x 3), washed with brine then dried over anhydrous $MgSO_4$, filtered, and purified by flash chromatography (10-30% EtOAc/hexanes) to afford an off white solid (1.4381 g, 83% yield).

4. Cost comparisons between oxidation catalyst

Table S12. Cost analysis of catalyst for oxidation

Cost per gram (USD)

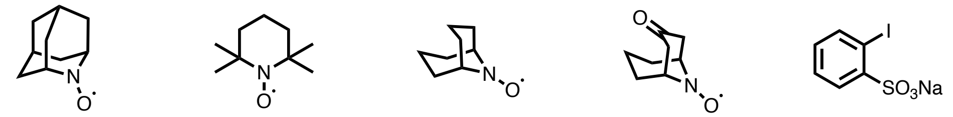

	**AZADO** CAS: 57625-08-8	**TEMPO** CAS:2564-83-2	**ABNO** CAS: 31785-68-9	**KETO-ABNO** CAS: 7123-92-4	**IBS precursor** CAS: 62973-69-7
PharmaBlock	$179	N/A	$106	$14	N/A
AmBeed	$413	$0.24	$106	$172	$23
TCI	$315	$5.2	N/A	N/A	$131
Sigma	$2,776	$6.76	$400	$301	$26.45
Combi-Blocks	N/A	$0.8	$105	$170	$30
Average cost/gram	$920.75	$3.25	$179.25	$164.25	$52.6

Note: The prices listed above are a comparison of the prices available to us on an academic level. The cost per gram was estimated based on the available prices. The cost per gram will vary depending on the size of the container purchased. On an industrial scale the cost per gram may differ. Worth noting, however, highly encouraged by the cost of TEMPO even at this scale it can serve as a potential and efficient alternative to AZADO for this oxidation.

5. HPLC data

HPLC: 1.5 mmol scale reaction of **11** to **6** prior to additional 0.1 equiv of NaOCl

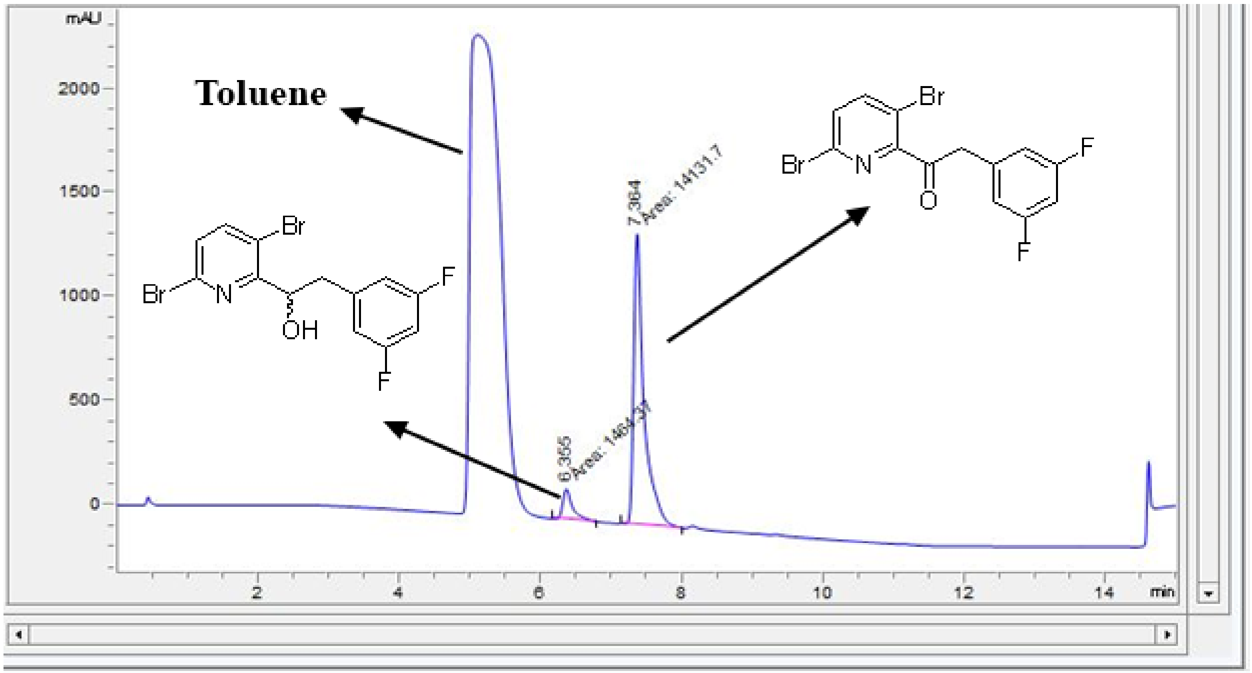

#	Time	Type	Area	Height	Width	Area%	Symmetry
1	6.355	MM	1464.4	145.7	0.1675	9.389	0.511
2	7.364	MM	14131.7	1397.9	0.1685	90.611	0.493

HPLC: 1.5 mmol scale reaction of **11** to **6**; after additional 0.1 equiv of NaOCl

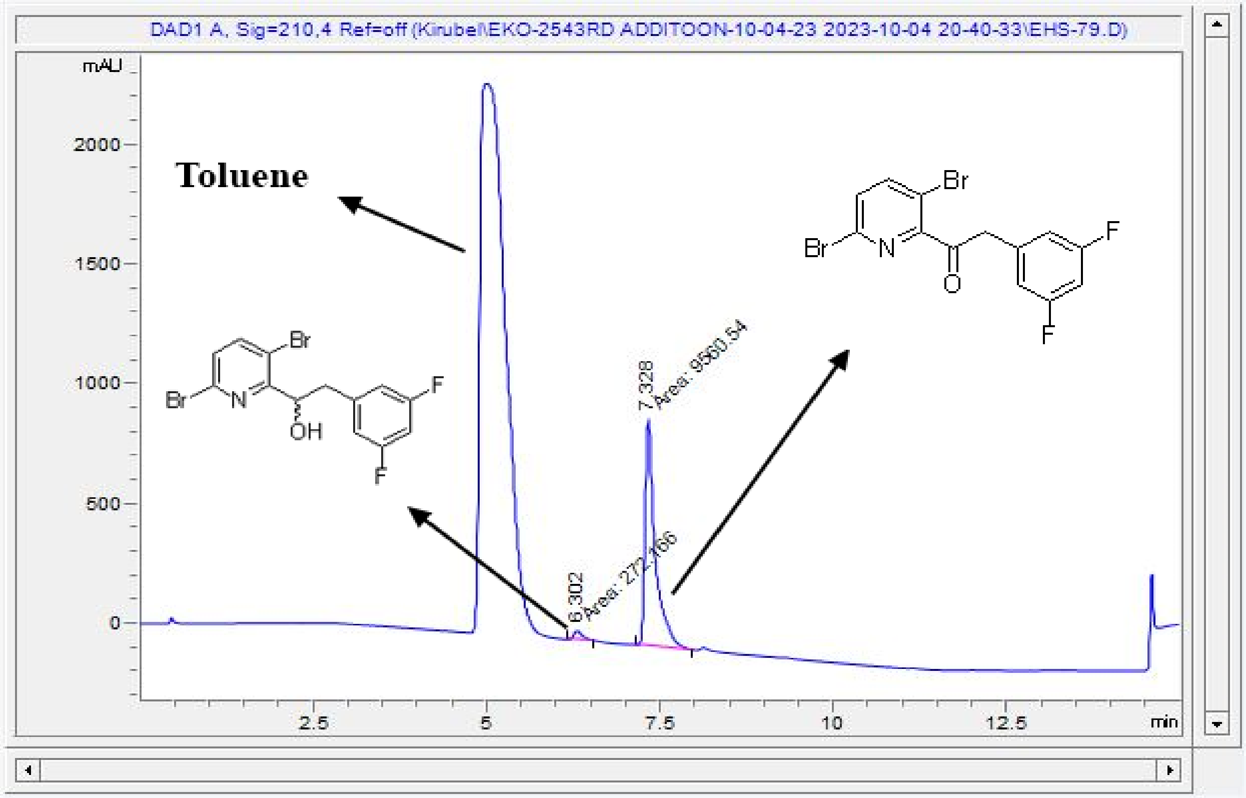

#	Time	Type	Area	Height	Width	Area%	Symmetry
1	6.302	MM	272.2	35.7	0.127	2.768	0.603
2	7.328	MM	9560.5	943.4	0.1689	97.232	0.47

HPLC: **6** after the recrystallization

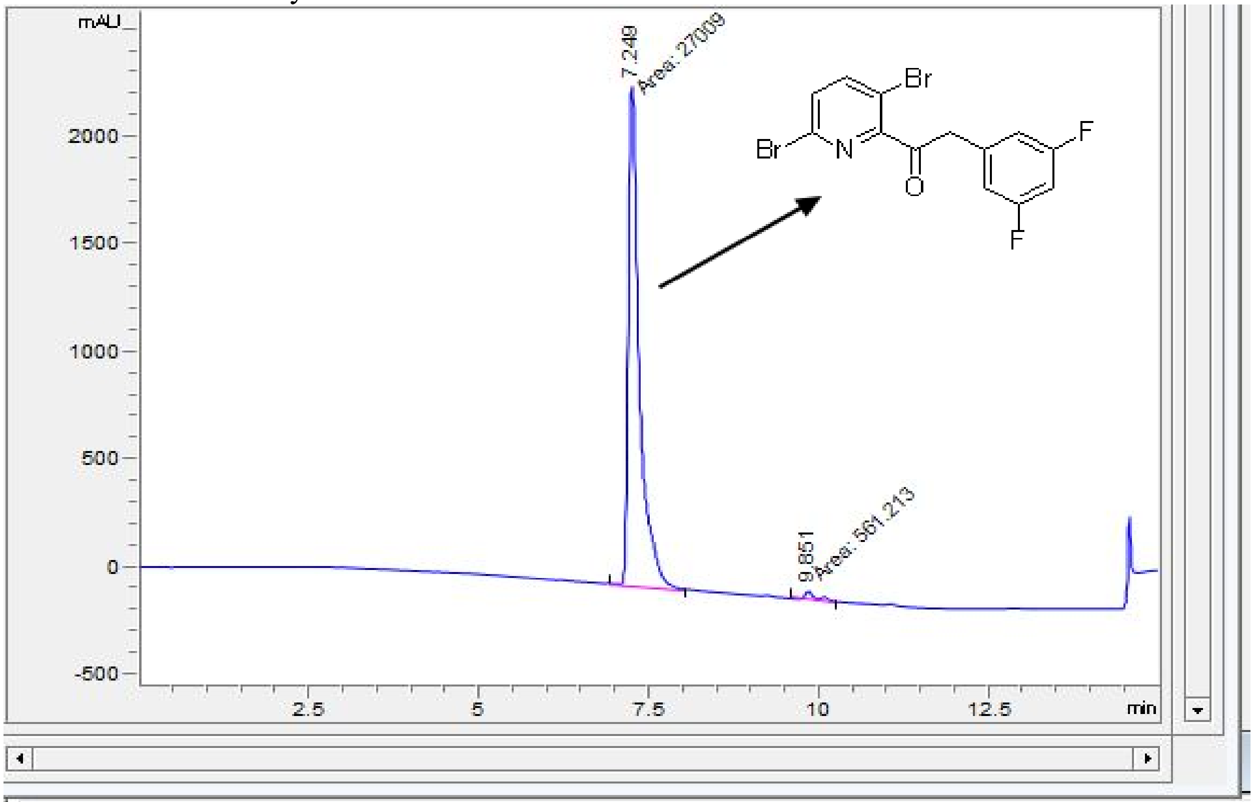

#	Time	Type	Area	Height	Width	Area%	Symmetry
1	7.249	MM	27009	2326.4	0.1935	97.964	0.476
2	9.851	MM	561.2	39.9	0.2343	2.036	0.237

HPLC: Initial reaction of recyclability study (Cycle 0)

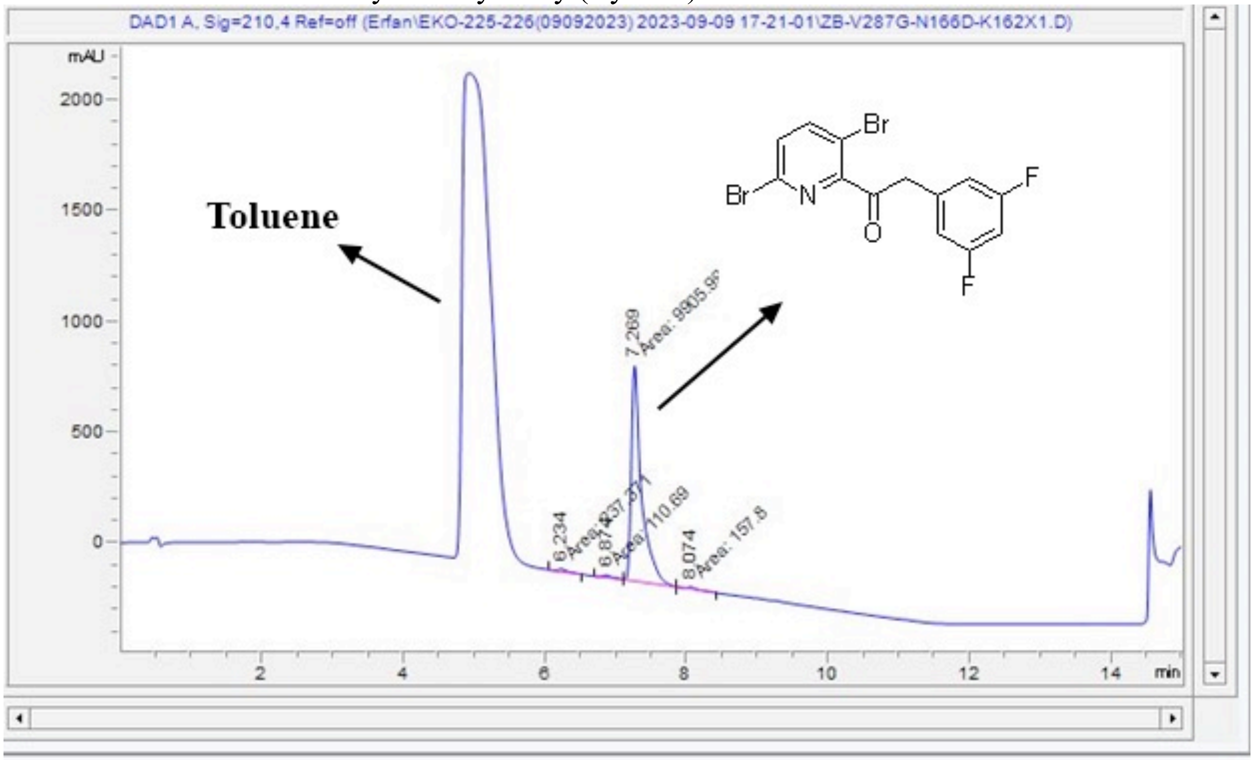

#	Time	Type	Area	Height	Width	Area%	Symmetry
1	6.234	MM	237.4	16.7	0.2372	2.280	0.646
2	6.874	MM	110.7	10.6	0.1733	1.063	0.343
3	7.269	MF	9906	975.5	0.1692	95.141	0.46
4	8.074	FM	157.8	12	0.2194	1.516	0.61

HPLC: Recyclability study; addition of 5 mol % TEMPO and 1.45 equivalents of NaOCl

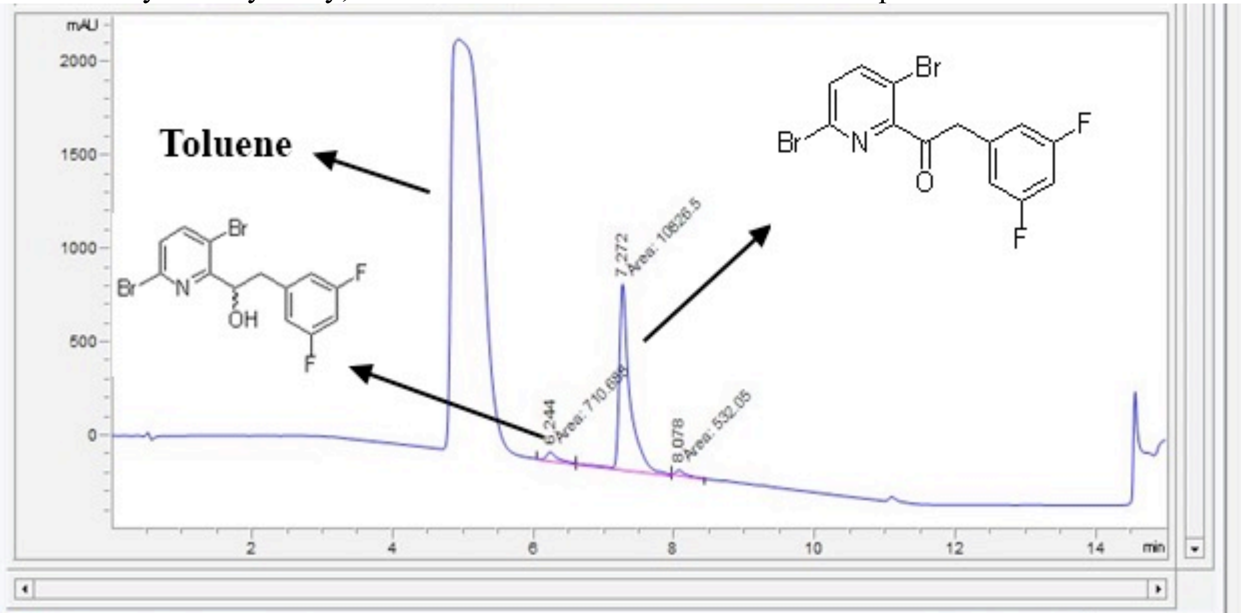

Opened Logbook File run.log

#	Time	Type	Area	Height	Width	Area%	Symmetry
1	6.244	MF	710.7	52.7	0.2248	5.888	0.488
2	7.272	MF	10826.5	998.2	0.1808	89.703	0.48
3	8.078	MM	532	38	0.2336	4.408	0.333

HPLC: Validation of HPLC results using qNMR to assess yield.

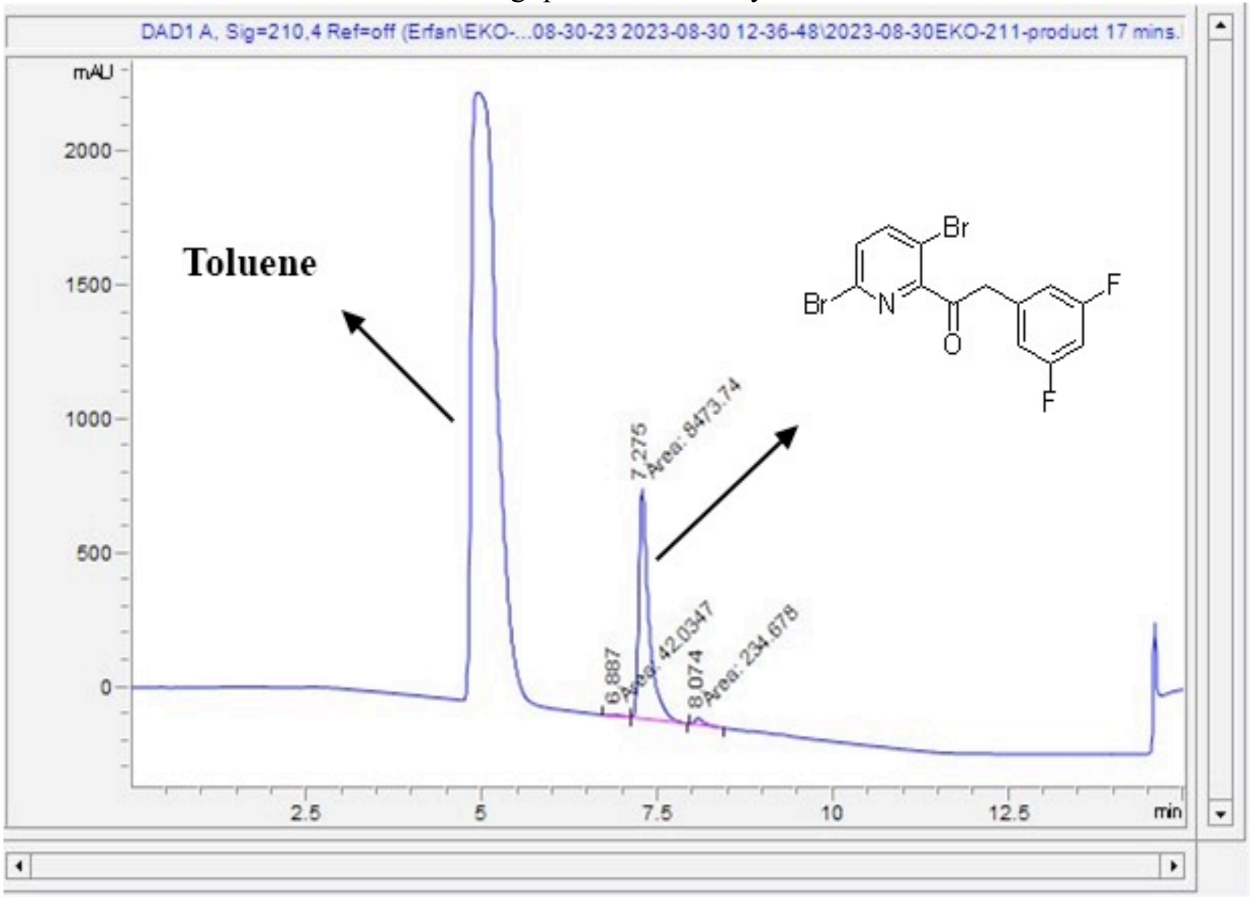

#	Time	Type	Area	Height	Width	Area%	Symmetry
1	6.887	MM	42	4.5	0.1556	0.480	0.463
2	7.275	MM	8473.7	855.8	0.165	96.838	0.49
3	8.074	MM	234.7	26	0.1504	2.682	0.481

6. References

1) Allan, K.M.; Batten, A.L.; Brizgys, G.; Dhar, S.; Doxsee I.J.; Goldberg, A.; Heumann, L.V.; Huang, Z. Kadunce, N.T.; Kazerani, S.; Willard, L.; NGO, V.X.; O'Keefe, B.M.; Rainey, T.J.; Roberts, B.J.;Shi, B.; Steinhuebel, D.P.; Tse W. C; Wagner, A.M.; Wang, X.; Wolckenhauer, S.A.; Wong, C.Y. Zhang, J.R. methods and intermediates for preparing a therapeutic compound useful ni the treatment of retroviridae vrial infection. WO2019/161280 A1. **2019**

2) Dussart-Gautheret, J.; Yu, T.; Ganesh, K.; Gaikwad Rajendra; Fabrice Gallou; Lipshutz, B. H. Impact of Aqueous Micellar Media on Biocatalytic Transformations Involving Transaminase (ATA); Applications to Chemoenzymatic Catalysis. *Green Chem*. **2022**, *24*, 6172–6178.

3) Lipshutz, B. H.; Hageman, M.; Fennewald, J. C.; Linstadt, R.; Slack, E.; Voigtritter, K. Selective Oxidations of Activated Alcohols in Water at Room Temperature. *Chem Commun*. **2014**, *50*, 11378–11381.

4) Thottumkara, A.P.; Bowsher, M.S.; Vinod, T.K. In Situ Generation of o-iodobenzoic Acid (IBX) and the Catalytic Use of It in Oxidation Reactions in the Presence of Oxone as a Co-oxidant. *Org. Lett*. **2005**, *7*, 2933-2936.

5) He, C.; Ma, F.; Zhang, W.; Tong, R. Reinvestigating $FeBr_3$-Catalyzed Alcohol Oxidation with H_2O_2: Is a High-Valent Iron Species (HIS) or a Reactive Brominatinf Species (RBS) Responsible for Alcohol Oxidation?. *Org. Lett*. **2022**, *24*, 3499-3503.

6) Graves, C. R.; Zeng, B.-S.; Nguyen, S. T. Efficient and Selective Al-Catalyzed Alcohol Oxidation via Oppenauer Chemistry. *J. Am. Chem. Soc*. **2006**, *128*, 12596–12597.

7) Uyanik, M.; Akakura, M.; Ishihara, K. 2-Iodoxybenzenesulfonic Acid as an Extremely Active Catalyst for the Selective Oxidation of Alcohols to Aldehydes, Ketones, Carboxylic Acids, and Enones with Oxone. *J. Am. Chem. Soc* **2008**, *131*, 251–262.

8) Lucio Anelli, P.; Biffi, C.; Montanari, F.; Quici, S. Fast and Selective Oxidation of Primary Alcohols to Aldehydes or to Carboxylic Acids and of Secondary Alcohols to Ketones Mediated by Oxoammonium Salts under Two-Phase Conditions. *J. Org. Chem*. **1987**, *52*, 2559–2562.

7. Analytical data

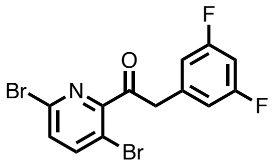

1-(3,6-Dibromopyridin-2-yl)-2-(3,5-difluorophenyl)ethan-1-one (**6**)

White solid. R_f:0.46 (20% EA/H)

^{1}H NMR (400 MHz, $CDCl_3$) δ 7.83 (d, *J* = 8.4 Hz, 1H), 7.47 (d, *J* = 8.3 Hz, 1H), 6.88 – 6.80 (m, 2H), 6.71 (m, 1H), 4.39 (s, 2H).

^{13}C{H} NMR (101 MHz, $CDCl_3$) δ 195.6, 163.0 (dd, *J* = 248.3, 12.9 Hz), 145.1, 139.3, 137.3 (t, *J* = 9.7 Hz), 131.9, 117.9, 113.4 – 112.7 (m), 102.7 (t, *J* = 25.2 Hz), 45.8.

^{19}F NMR (376 MHz, $CDCl_3$) δ -109.98

HRMS (ESI-TOF) *m/z*: $[M-H]^-$ calcd for $C_{13}H_6Br_2F_2NO$: 387.8784; found 387.8791.

mp 62-65 °C

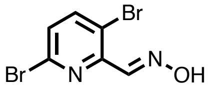

(*E*)-3,6-Dibromopicolinaldehyde oxime (**8**)

Light yellow solid. R_f: 0.43 (30% EtOAc/hexanes)

^{1}H NMR (400 MHz, DMSO-d_6) δ 11.69 (s, 1H), 8.08 (d, *J* = 8.5 Hz, 1H), 7.67 (s, 1H), 7.61 (d, *J* = 8.5 Hz, 1H).

^{13}C{H} NMR (101 MHz, DMSO-d_6) δ 151.7, 143.2, 143.0, 139.1, 129.3, 119.4.

Spectral data matches those reported in the literature[1]

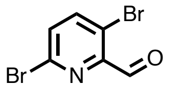

3,6-Dibromopicolinaldehyde (**9**)

Pale yellow/golden solid. R_f:0.33 (20% EtOAc/hexanes)

^{1}H NMR (400 MHz, DMSO-d$_6$) δ 9.95 (s, 1H), 8.21 (dd, J = 8.4, 1.2 Hz, 1H), 7.85 (dd, J = 8.4, 1.2 Hz, 1H).

^{13}C{H} NMR (101 MHz, DMSO-d$_6$) δ 189.4, 148.6, 145.7, 140.2, 133.2, 120.3.

Spectral data matches those reported in the literature[1]

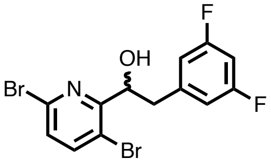

1-(3,6-Dibromopyridin-2-yl)-2-(3,5-difluorophenyl)ethan-1-ol (**11**)

White solid. R_f:0.39 (20% EtOAc/hexanes)

^{1}H NMR (400 MHz, CDCl$_3$) δ 7.72 (d, J = 8.3 Hz, 1H), 7.33 (d, J = 8.2 Hz, 1H), 6.81 – 6.71 (m, 2H), 6.68 (tt, J = 9.0, 2.3 Hz, 1H), 5.16 (dd, J = 8.3, 3.4 Hz, 1H), 3.78 (s, 1H), 3.12 (dd, J = 13.8, 3.4 Hz, 1H), 2.82 (dd, J = 13.8, 8.3 Hz, 1H).

^{13}C{H} NMR (126 MHz, CDCl$_3$) δ 162.9 (dd, J = 248.0, 13.0 Hz), 160.2, 143.0, 141.4 (t, J = 9.2 Hz), 139.9, 128.7, 118.0, 112.4 (dd, J = 19.3, 5.5 Hz), 102.2 (t, J = 25.3 Hz), 72.1, 43.1 (t, J = 2.0 Hz).

^{19}F NMR (471 MHz, CDCl$_3$) δ -110.51.

HRMS (ESI-TOF) *m/z*: $[M+H]^+$ calcd for $C_{13}H_{10}Br_2F_2NO$: 391.9097; found 391.9100.

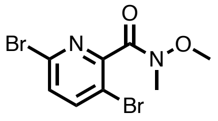

3,6-Dibromo-*N*-methoxy-*N*-methylpicolinamide (**15**)

Off white solid. R_f:0.35 (30% EtOAc/hexanes)

^{1}H NMR (400 MHz, CDCl$_3$) δ 7.74 (d, J = 8.4 Hz, 1H), 7.39 (d, J = 8.4 Hz, 1H), 3.61 (s, 3H), 3.37 (s, 2H). (Mixture of rotamers)

^{13}C{H} NMR (126 MHz, $CDCl_3$) δ 165.7, 154.6, 142.5, 139.7, 129.6, 116.4, 61.7, 32.0. (Mixture of rotamers)

HRMS (ESI-TOF) *m/z*: $[M+H]^+$ calcd for $C_8H_9Br_2N_2O_2$: 322.9031;found 322.9033.

8. NMR Spectra

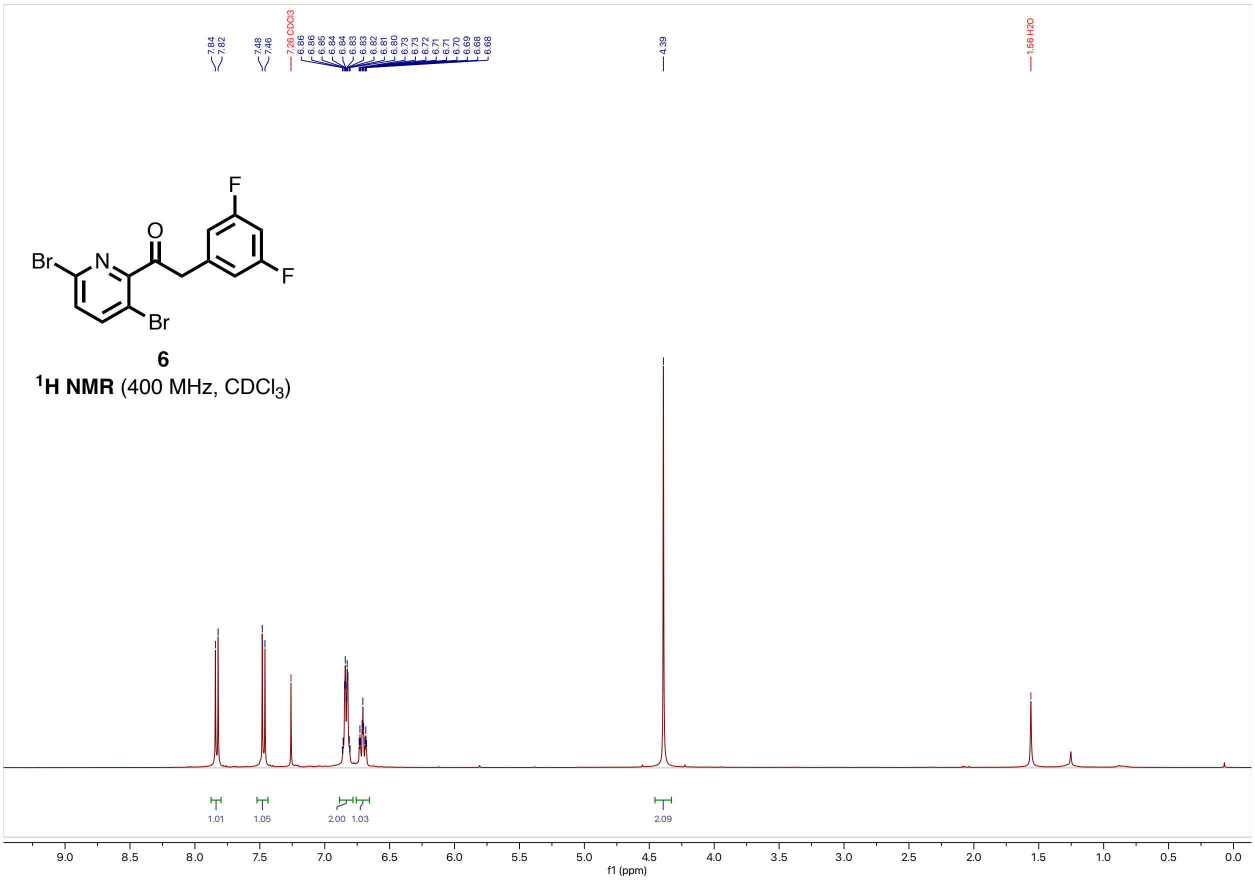

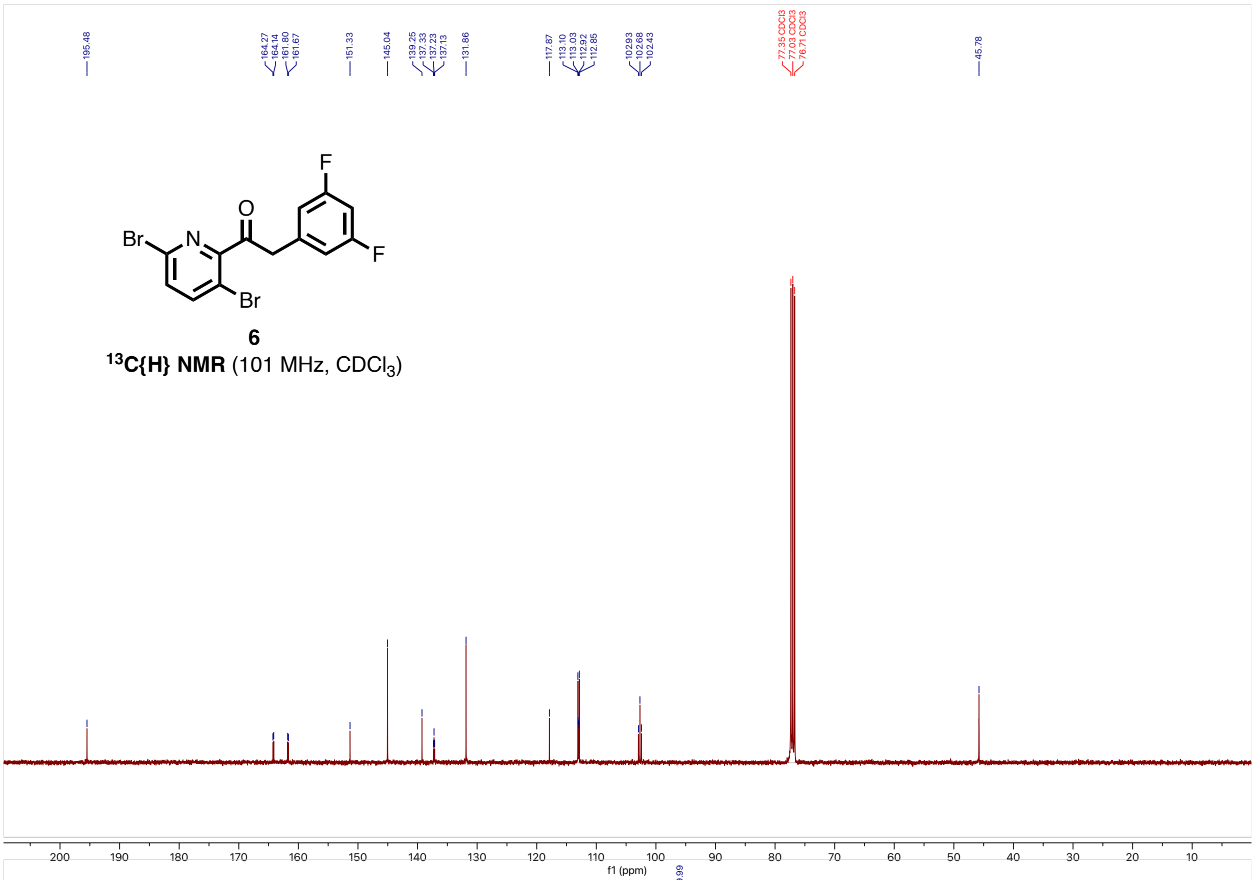
195.48
164.27
164.14
161.80
161.67
151.33
145.04
139.25
137.33
137.23
137.13
131.86
117.87
113.10
113.03
112.92
112.85
102.93
102.68
102.43
77.35 CDCl3
77.03 CDCl3
76.71 CDCl3
45.78
Br
N
O
F
F
Br
6
^{13}C{H} NMR (101 MHz, $CDCl_3$)
f1 (ppm)

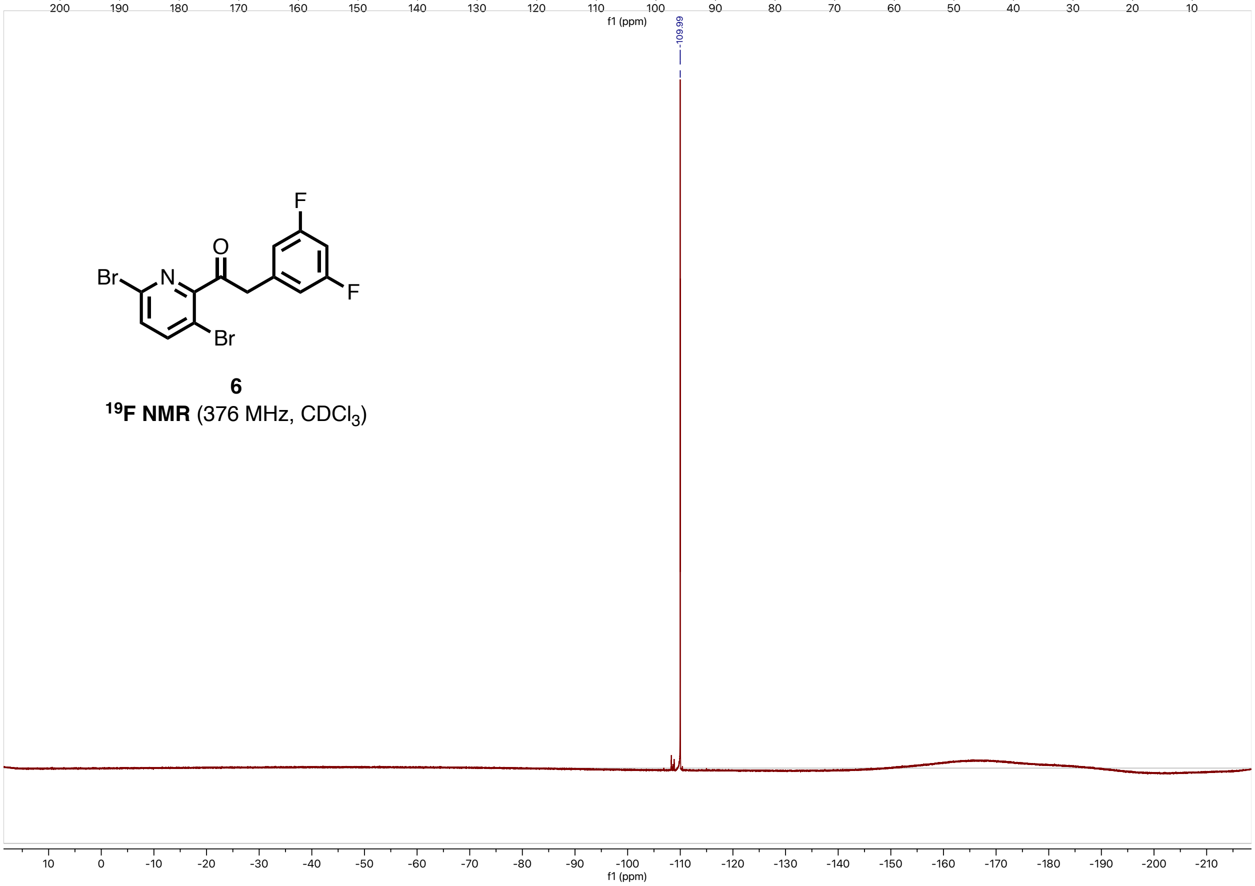
-109.99
Br
N
O
F
F
Br
6
^{19}F NMR (376 MHz, $CDCl_3$)
f1 (ppm)

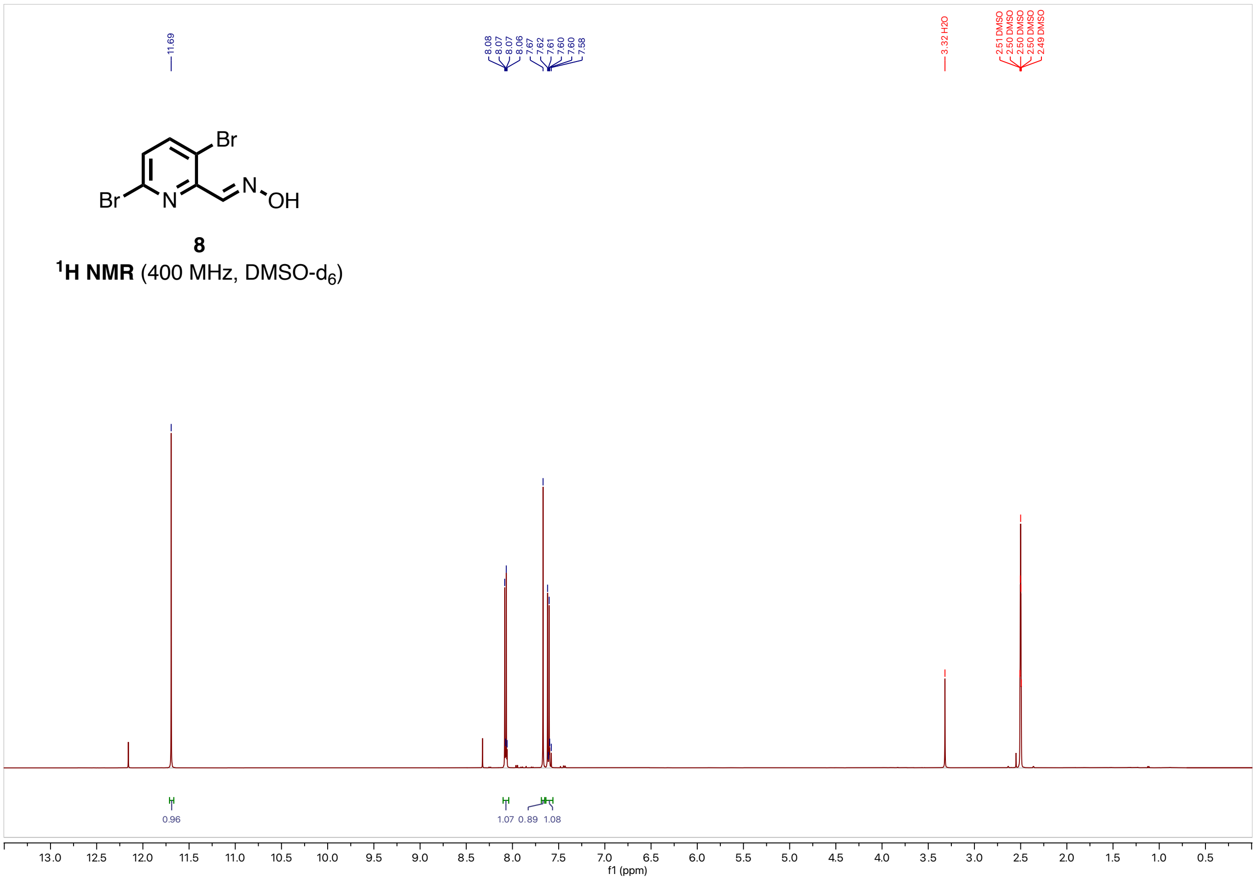

8

^{1}H NMR (400 MHz, DMSO-d_6)

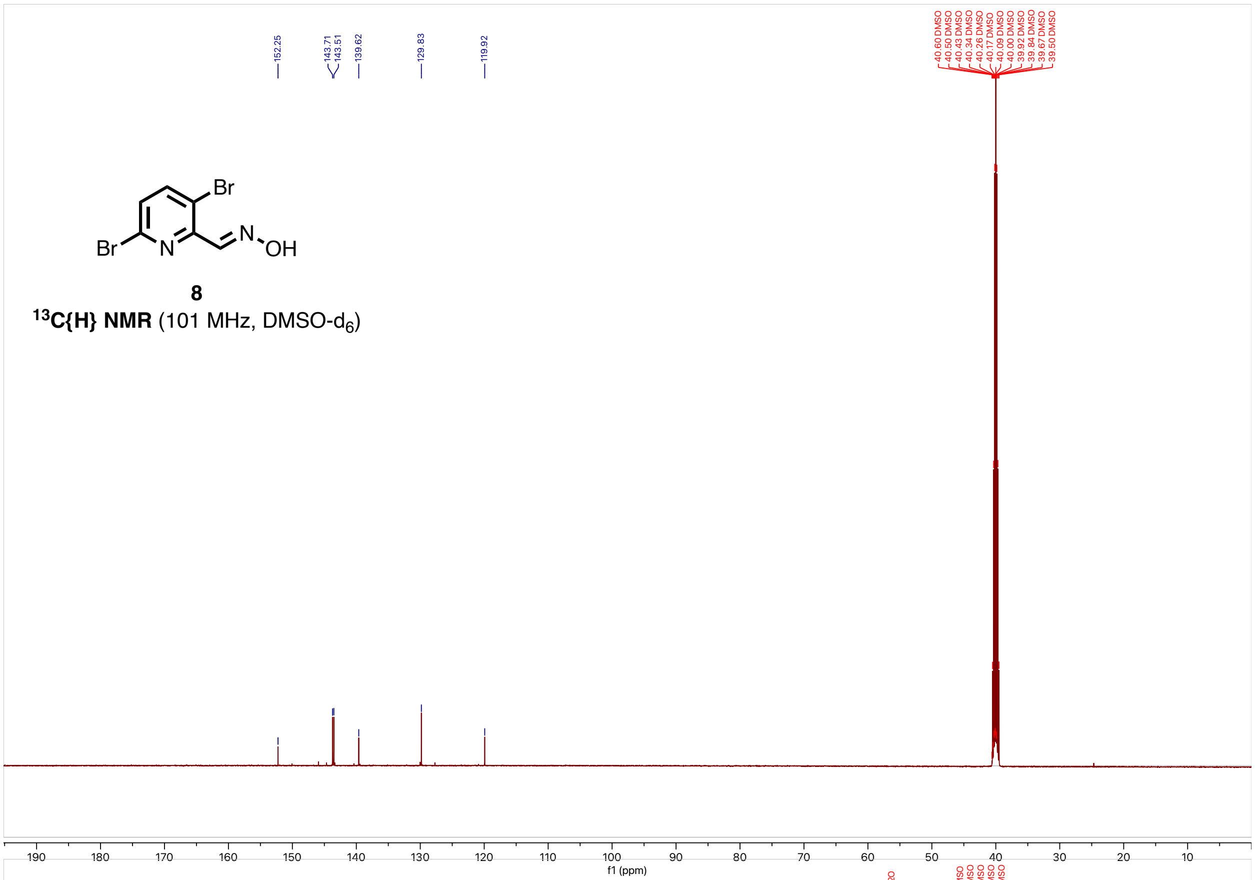
152.25
143.71
143.51
139.62
129.83
119.92
40.60 DMSO
40.50 DMSO
40.43 DMSO
40.34 DMSO
40.26 DMSO
40.17 DMSO
40.09 DMSO
40.00 DMSO
39.92 DMSO
39.84 DMSO
39.67 DMSO
39.50 DMSO
Br
Br
N
N
OH
8
13C{H} NMR (101 MHz, DMSO-d6)
190 180 170 160 150 140 130 120 110 100 90 80 70 60 50 40 30 20 10
f1 (ppm)

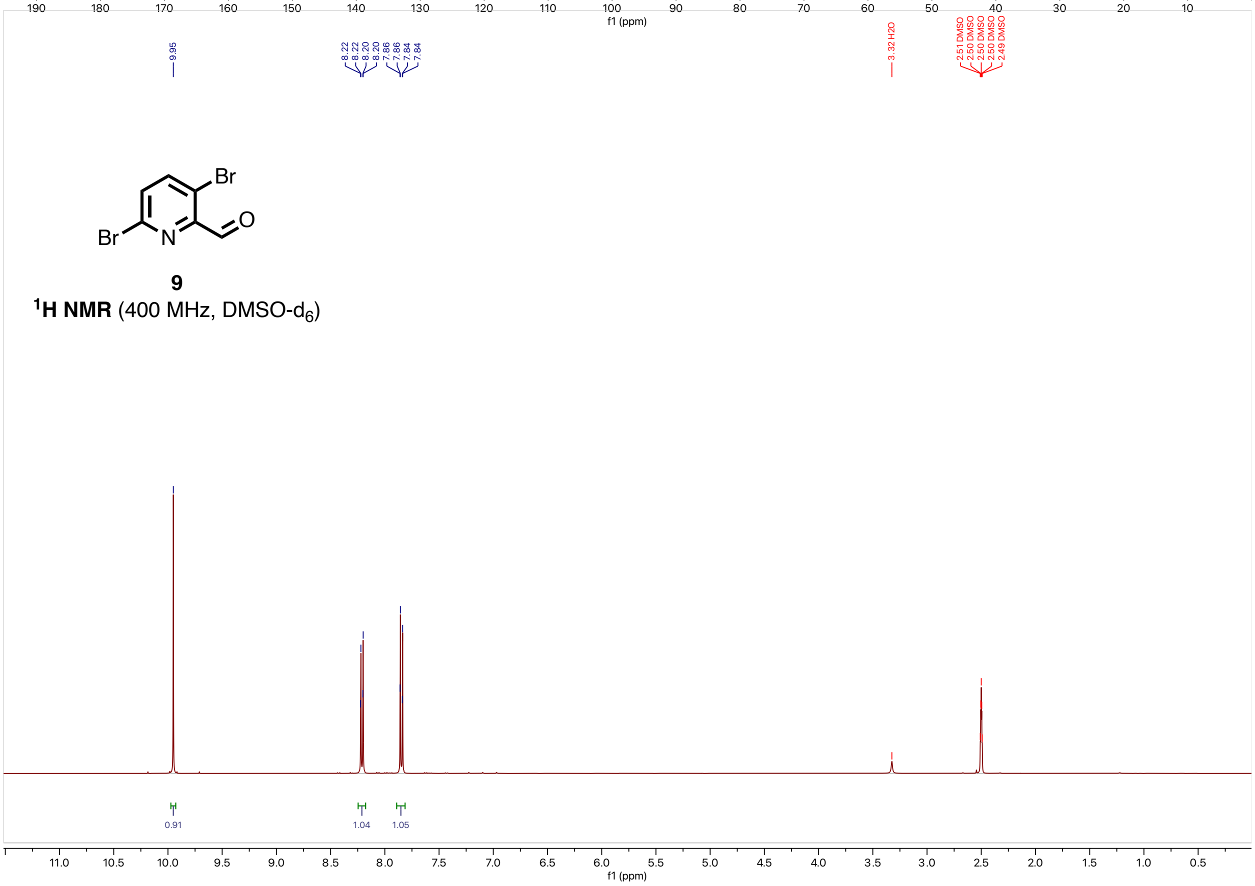
9.95
8.22
8.22
8.20
8.20
7.86
7.86
7.84
7.84
3.32 H2O
2.51 DMSO
2.50 DMSO
2.50 DMSO
2.50 DMSO
2.49 DMSO
Br
Br
N
O
9
1H NMR (400 MHz, DMSO-d6)
0.91
1.04
1.05
11.0 10.5 10.0 9.5 9.0 8.5 8.0 7.5 7.0 6.5 6.0 5.5 5.0 4.5 4.0 3.5 3.0 2.5 2.0 1.5 1.0 0.5
f1 (ppm)

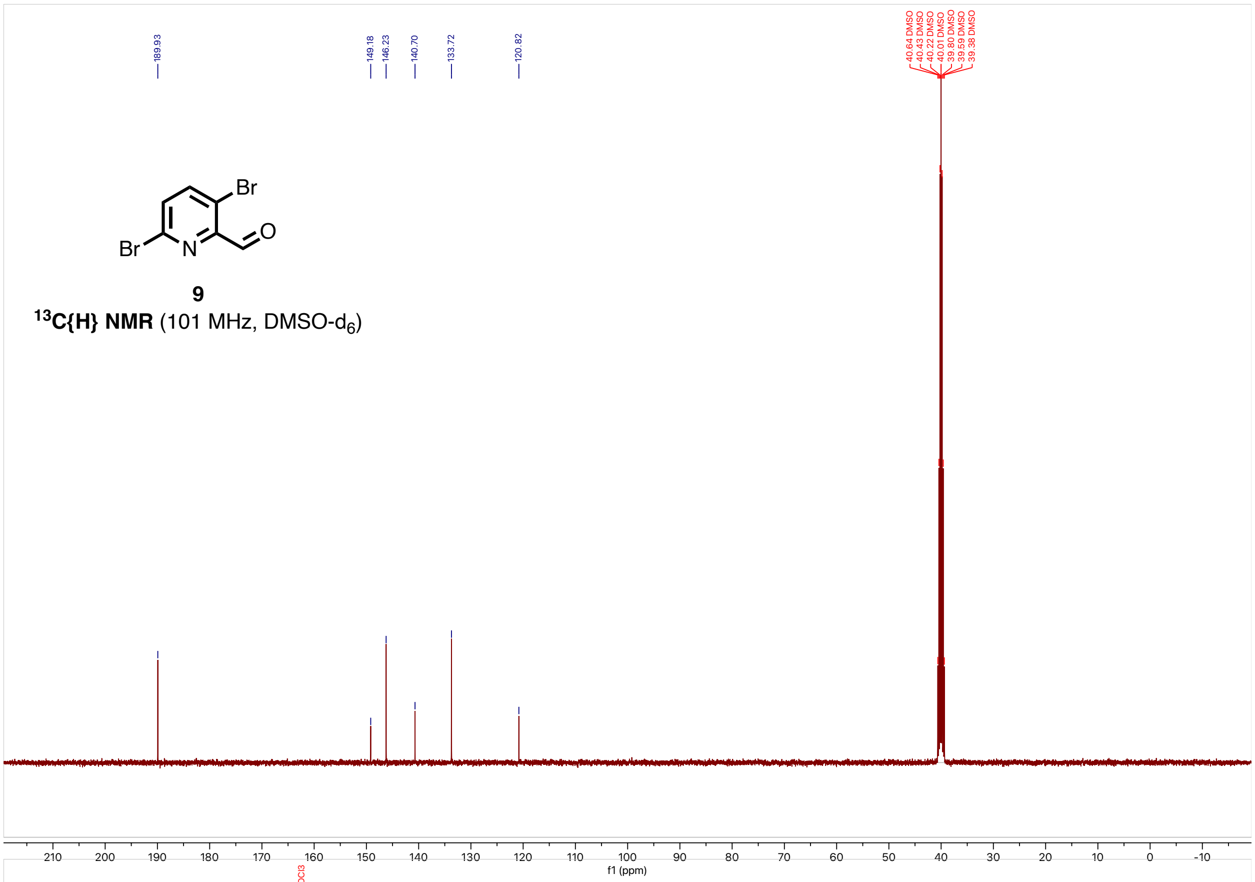
189.93
149.18
146.23
140.70
133.72
120.82
40.64 DMSO
40.43 DMSO
40.22 DMSO
40.01 DMSO
39.80 DMSO
39.59 DMSO
39.38 DMSO
Br
Br
N
O
9
13C{H} NMR (101 MHz, DMSO-d6)
f1 (ppm)

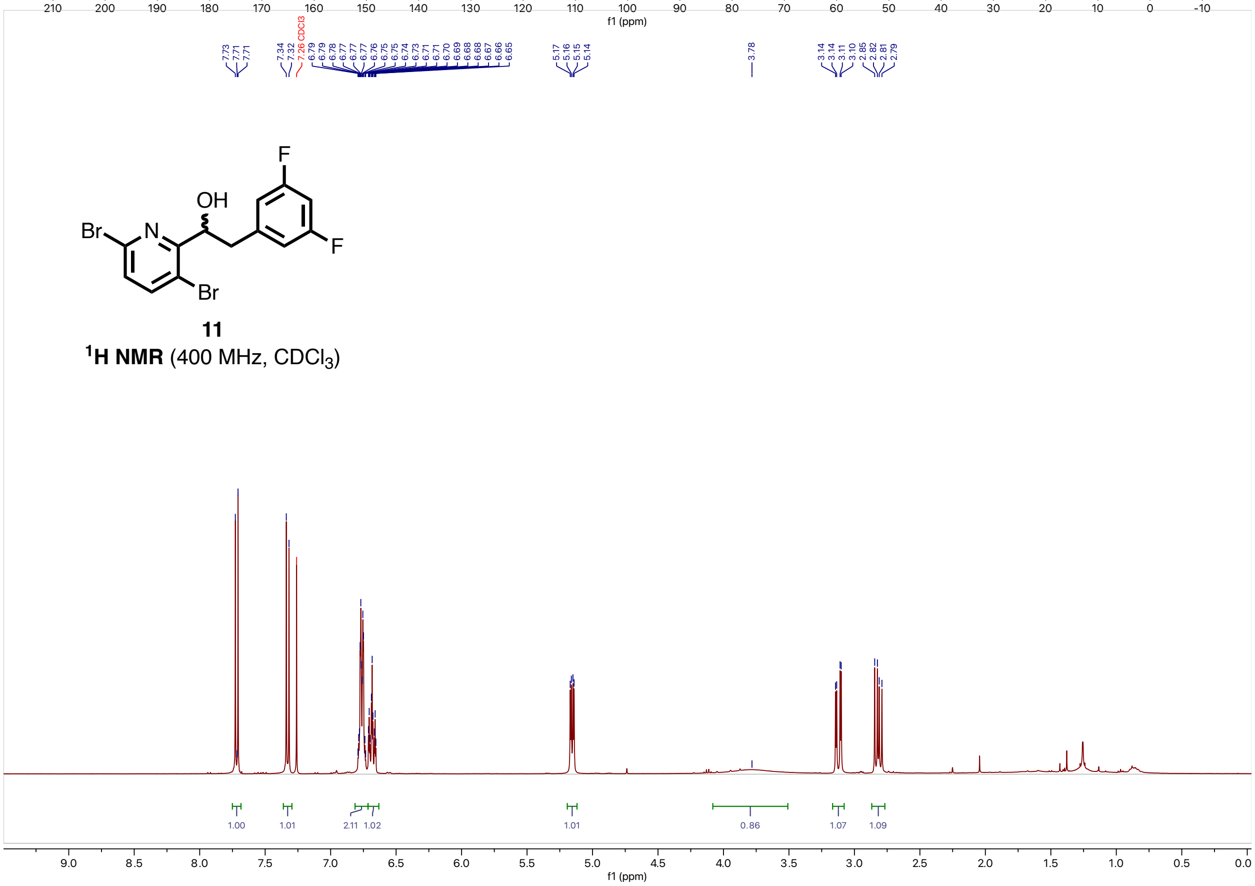
7.73
7.71
7.71
7.34
7.32
7.26 CDCl3
6.79
6.79
6.78
6.77
6.77
6.77
6.76
6.75
6.75
6.74
6.73
6.71
6.71
6.70
6.69
6.68
6.68
6.67
6.66
6.65
5.17
5.16
5.15
5.14
3.78
3.14
3.14
3.11
3.10
2.85
2.82
2.81
2.79
F
OH
Br
N
F
Br
11
1H NMR (400 MHz, CDCl3)
1.00
1.01
2.11
1.02
1.01
0.86
1.07
1.09
f1 (ppm)

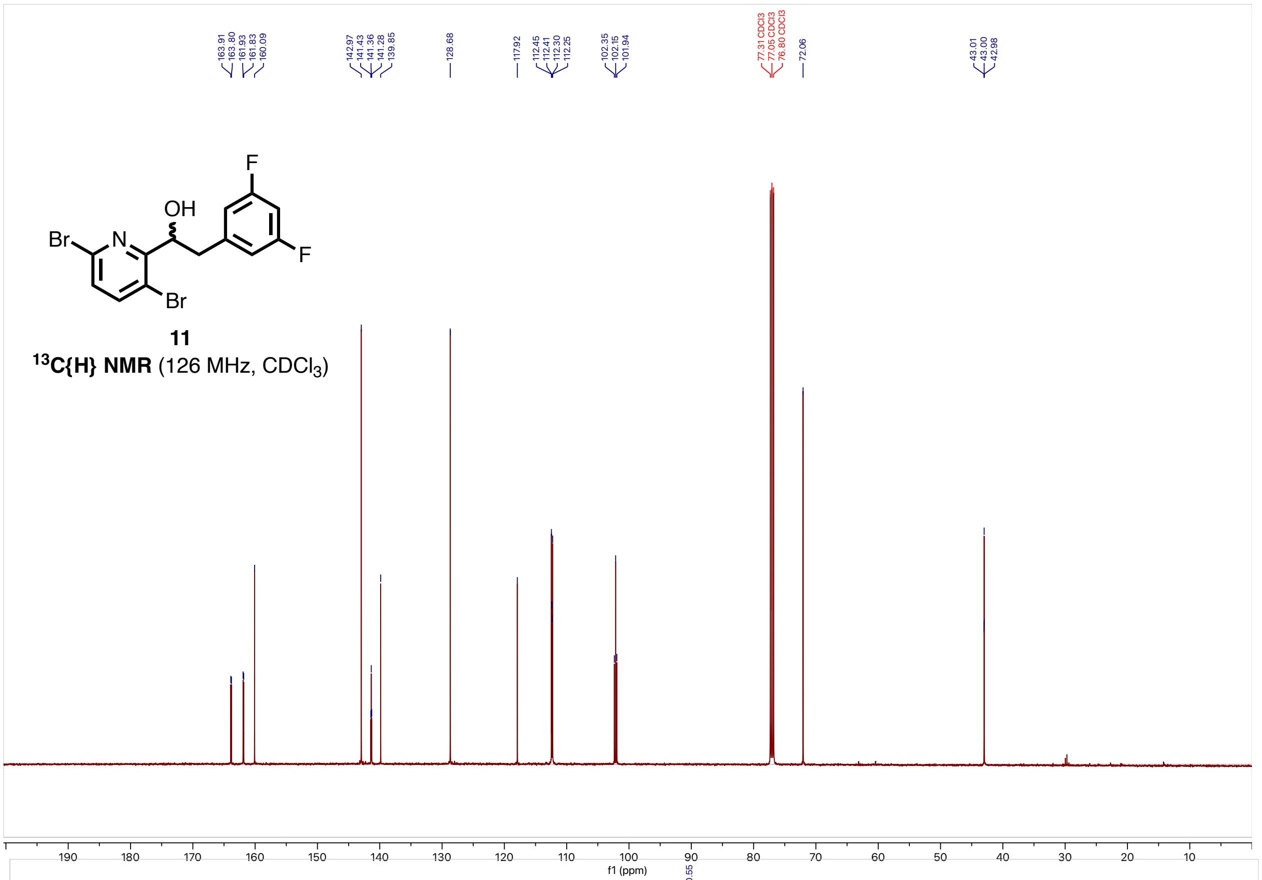

11

^{13}C{H} NMR (126 MHz, $CDCl_3$)

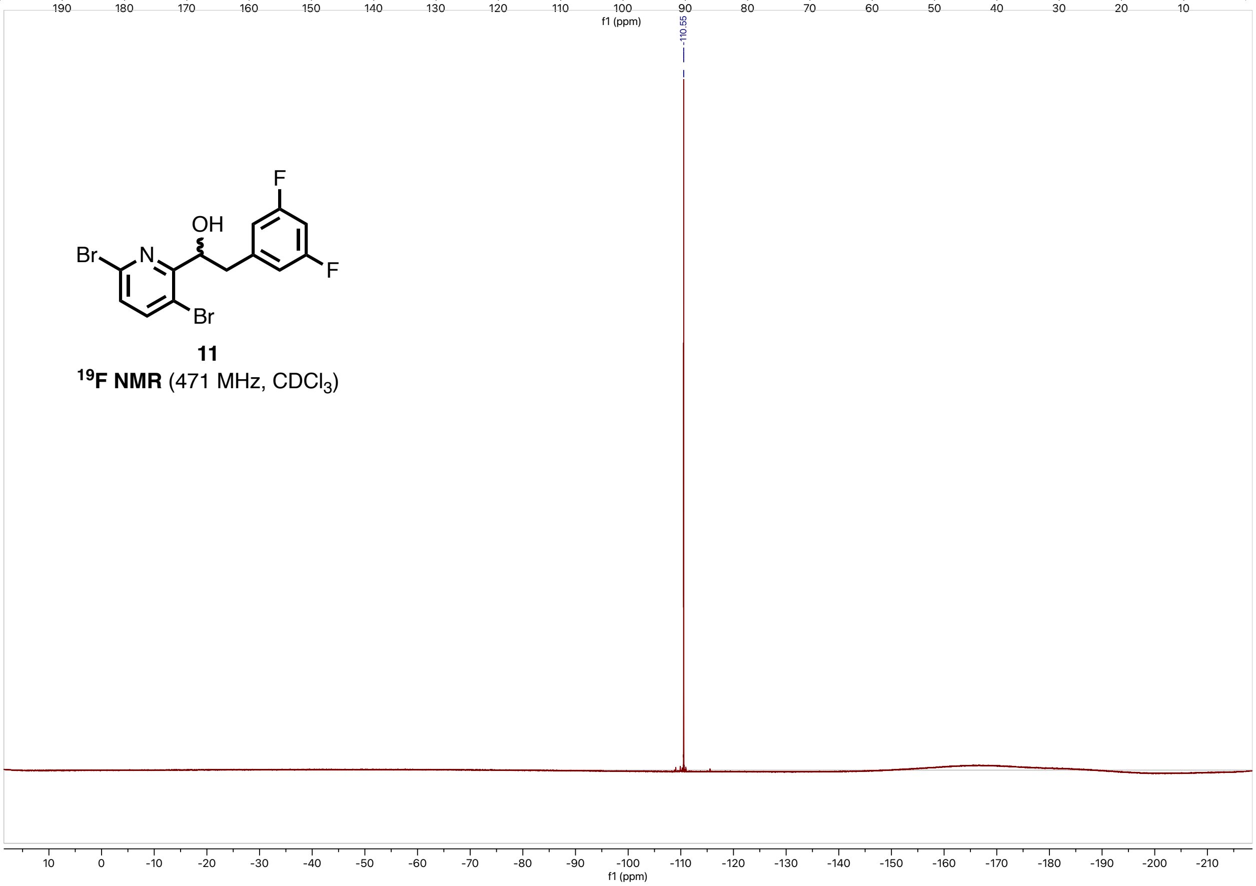

11

^{19}F NMR (471 MHz, $CDCl_3$)

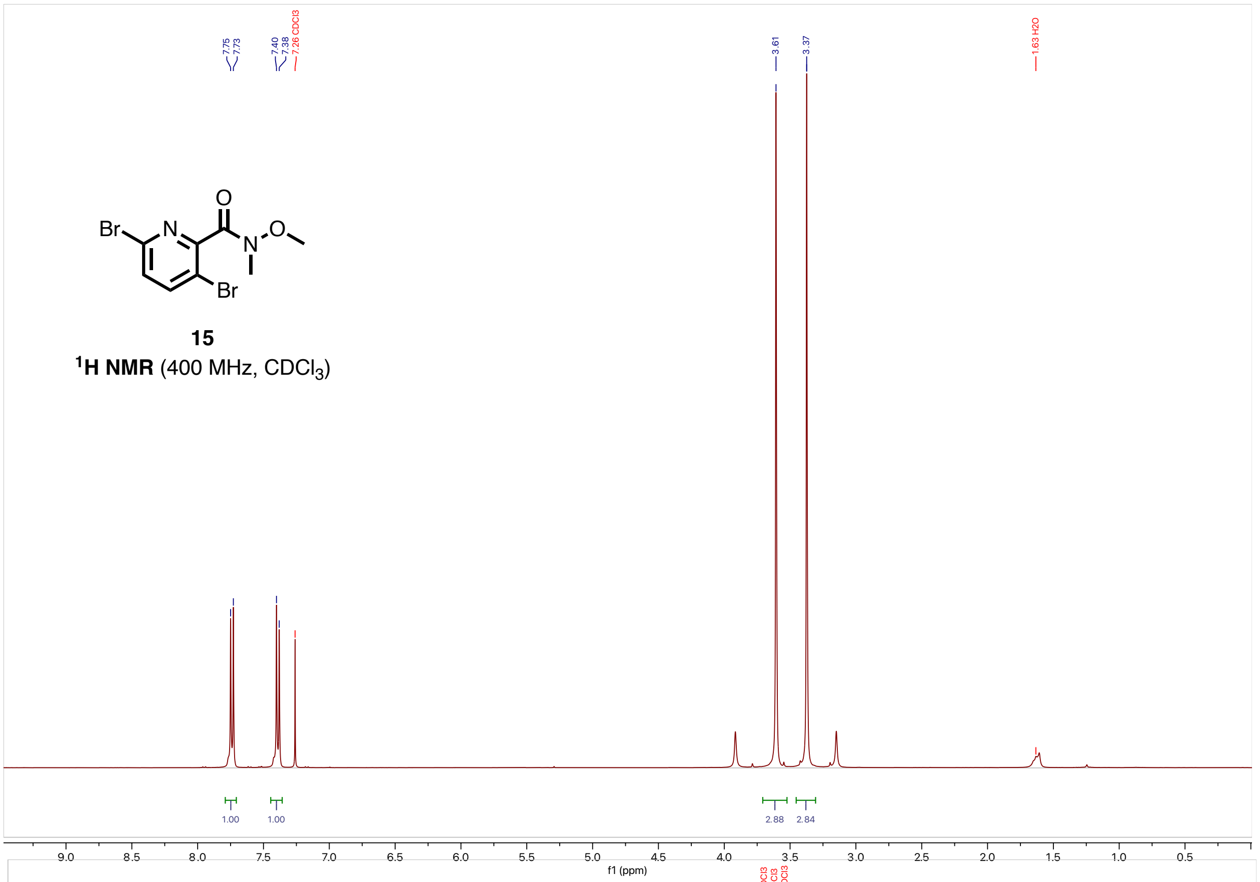
7.75
7.73
7.40
7.38
7.26 CDCl3
3.61
3.37
1.63 H2O
Br
N
O
N
O
Br
15
1H NMR (400 MHz, CDCl3)
1.00
1.00
2.88
2.84
f1 (ppm)

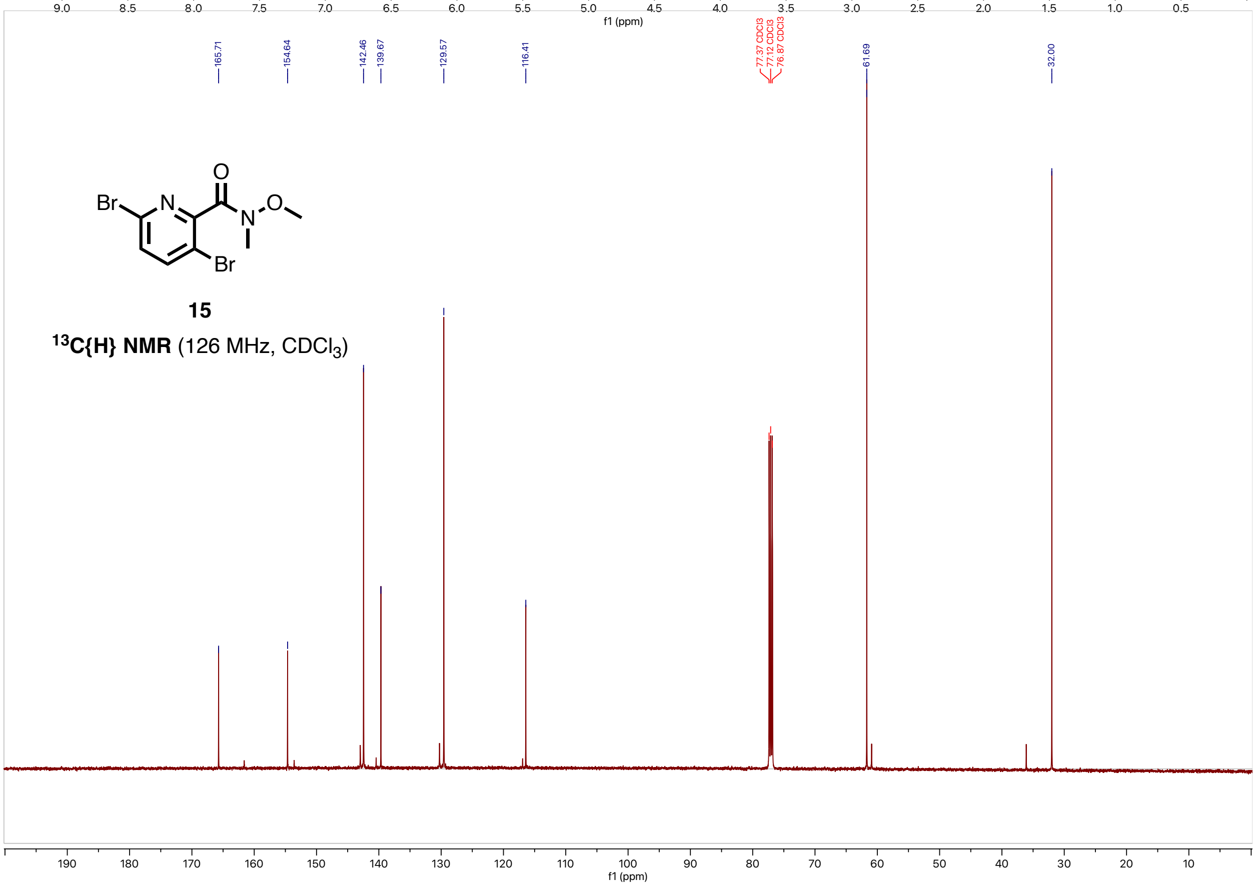
165.71
154.64
142.46
139.67
129.57
116.41
77.37 CDCl3
77.12 CDCl3
76.87 CDCl3
61.69
32.00
Br
N
O
N
O
Br
15
13C{H} NMR (126 MHz, CDCl3)
f1 (ppm)
